# Challenges in Analyzing the Biological Effects of Resveratrol

**DOI:** 10.3390/nu8060353

**Published:** 2016-06-09

**Authors:** Cihan Suleyman Erdogan, Ole Vang

**Affiliations:** Department of Science and Environment, Roskilde University, Universitetsvej 1, Roskilde DK-4000, Denmark; cihan@ruc.dk

**Keywords:** resveratrol, combinatory effect, complex mixtures, bioavailability, preventive human studies

## Abstract

The suggested health effects (e.g., disease prevention) of dietary bioactive compounds such as resveratrol are challenging to prove in comparison to man-made drugs developed for therapeutic purposes. Dietary bioactive compounds have multiple cellular targets and therefore have a variety of biological effects. Extrapolating the biological effects of dietary compounds from *in vitro* and *in vivo* animal experiments to humans may lead to over- or under-estimation of the effect and role of these compounds. The present paper will discuss a few of these challenges and suggest directions for future research. Questions we address include: (1) Is the combinatorial effect of resveratrol and other compounds real? (2) What are the real and relevant doses of resveratrol after administration? and (3) Is it possible to estimate the preventive effect of resveratrol by clinical trials using standard experimental designs? The examples concerning resveratrol taken from the scientific literature are mainly from 2010 and later. The challenges pointed out in this review are similar to most naturally occurring bioactive compounds.

## 1. Introduction

There is a high degree of interest in dietary bioactive compounds found in, e.g., wine (as resveratrol (Resv)) or chocolate (various flavonols). The attraction is caused by the claimed health-promoting activity of the compounds and/or the mixtures they are a part of. Such claims are based on either *in vitro* studies estimating the biological activity of single compounds or mixtures analyzing a single biomarker, or from animal studies using pure compounds or mixtures in relatively high amounts during a short period of exposure. Support is also in several cases derived from epidemiological studies where the relation between dietary intake of specific food items to a specific disease has been investigated. One epidemiological study did not show a clear health-promoting effect related to the intake of dietary Resv [1], a conclusion questioned by Brown *et al.* [2].

The proof of a biological response of dietary components has to rely on all three types of studies (mechanistic studies (*in vitro*), animal studies, and clinical studies); none of these can stand alone. Many of the postulated health-promoting effects of dietary compounds besides macromolecules, fiber, and vitamins are therefore not very well-documented, which creates a lot of confusion in society concerning their biological effects. The lack of strong documentation is not only caused by a lack of research, but also because there are some structural challenges in proving the health-promoting effects of dietary substances, as they act differently compared with man-made drugs.

The present review will discuss the challenges involved in proving the health-promoting effects of dietary components by using Resv as a case story. From the first identification of Resv in the *Veratrum grandiflorum* plant back in 1939 by Takaoka [3] to the present, more than 8000 scientific articles have covered different aspects of the biological effects of Resv.

To discuss the challenges of analyzing the biological activity of Resv, we want to address these questions: (1) What is the combinatory effect of resveratrol and other compounds? (2) What are the real and relevant doses of resveratrol? and (3) Are preventive clinical trials a possibility?

The challenge of studying the biological effects of Resv in combination with other compounds (dietary components or drugs) is two-faced: As Resv is only present in low amounts in the normal human diet, one has to ask whether Resv has any relevant biological effect in humans where the exposure to the compound is life-long. The biological activities are only relevant if Resv acts in combination with other dietary compounds acting in the same line following life-long exposure. Secondly, drug–drug interactions have been observed with Resv [4], and the number of examples for a potentiation of the response of a given drug by Resv is increasing.

A second challenge we have identified is the fact that the bioavailability of resveratrol seems to be rather low because of a fast metabolism. However, at the same time, not all of the administered Resv can be accounted for. About 20%–30% of the Resv is not recovered in urine or feces [5], so one may ask whether Resv is still in the cells in different parts of the body. It is possible that Resv is associated with lipid compartments and released slowly.

Lastly, in the last five years, several clinical trials have been released where Resv has been administrated at a relatively high dose, for a short time, to test the reduction of disease-related biomarkers. In relation to the effect of Resv on diabetes, only diabetic patients had beneficial effects from Resv treatment, a recent meta-analysis showed [6]; there were no visible effects in healthy subjects. Is it possible to conduct a clinical study proving or disproving the disease-preventative effect of Resv? Based on the present data, we will suggest some directions for future research to solve this challenge.

To reduce the amount of data included here, we mainly include data published after 2010.

## 2. The Combinatory Effect—A Boosted Effect?

There are many claims in the scientific literature showing combinatory, additive, or synergistic effects. One has to consider this carefully, as we are determining the responses of several compounds at the same time. Wine is not Resv-only and the effect of Resv in the wine may be different from the effect of Resv alone. The awareness of diet–drug interaction is increasing, whereas drug–drug interaction has been the focus for many years. Considering the naturally occurring compounds with weak affinities present in mixtures of many bioactive compounds, the picture becomes confusing. Extrapolating the effect of short-term exposure to the pure compound at high doses to life-long exposure at low doses in combination with various other bioactive compounds is a difficult task. Chou suggested a model for the combination of two compounds to identify whether the interaction is additive, synergistic, or antagonistic [7]. New and better mathematical models have to be established to analyze the combined effect of multiple compounds, but this has to rely on knowledge of the effects of combinations of several compounds. These mixtures need to be characterized according to exact composition when used for experiments. Different examples of interaction with Resv will be discussed.

Studies where the focus is on Resv (as an anti-carcinogen) together with a carcinogen also investigate a form of drug–drug interaction, but they are outside the scope of the present review.

### 2.1. Drug–Drug Interaction with Resveratrol

The combinatory effect of Resv and other chemicals that the human body is exposed to is therefore important to be aware of, as this response may be quite different from the effect observed for a single compound, either the drug or Resv. Often, scientists are studying the effects of the single compounds in separate experiments and one will not get any information on their interactions. One such known interaction of Resv, because of the modulation of xenobiotic metabolism and cellular transporting systems, has been reviewed recently [8]. It was shown that various isoforms of the Cytochrome P450 1–3 families and uridine-glucuronosyltransferase are modulated by Resv. Therefore, intake of Resv together with a given drug will change the pharmacokinetic of the drug, which is indeed observed in human studies [4]. One has to consider that the study of Chow *et al.* used a dose of 1 g of Resv daily for a four-week exposure, a level of exposure humans never ordinarily reach through dietary Resv intake in the form of wine, peanuts, berries, *etc.* (2–5 mg), but the study showed the modulatory potential of Resv and similar compounds in the diet. Further investigations have to focus on whether similar effects may be obtained when humans are exposed to various Cytochrome P450 and phase 2 enzyme activity inhibitors at low doses in combination in the diet.

Besides the modulation of metabolism and/or drug transportation, substances like Resv may have a great impact on other drug targets. In other words, Resv may potentiate or reduce the effect of a drug. Here we include data from *in vitro* and animal studies from the past five years. The metabolism of Resv or the drugs is not included in the equation in most of the *in vitro* models described below. Therefore, translation to the clinic should be done with care.

Cisplatin, carboplatin, and oxaliplatin are well-used chemotherapy drugs because they crosslink DNA in fast-growing cells. In most of the experiments, an additive effect and sometimes a synergistic effect has been observed in the reduction of the cell viability of various cancer cell lines [9,10]. Similar effects were observed with DNA interchelating drugs like doxorubicin and docetaxel [11], toposimerase inhibitors (like etoposide) [12], and nucleotide analogs (fluorouracil, fludarabine, cladribine, gemcitabine, clofarabine, and decitabine) [13]. Also, the combination of Resv with DNA-alkylating substances (cyclophosphamide, temozolomide, melphalan, and carmustine) caused a potentiation compared with the effect of the drug alone [14]. Reduced or counteracting effects of Resv have been observed as well, when Resv is combined with inhibitors of microtubules (vinblastine and paclitaxel), depending on the order of treatment [9]. Similarly, the cytotoxic effect of the proteasome inhibitor MG132 is reduced by Resv [15]. The full list of publications is found in Table S1. In several cases, a synergistic effect is postulated, without a clear documentation for synergism, e.g., [16,17].

Formation of diethylstilbestrol–DNA adducts, as a side effect of hormone treatment, is reduced by concomitant treatment with Resv [18] and EGFR inhibition by gefitinib [19], and the apoptosis-inducing effect of arsenic trioxide is potentiated by Resv [20] (Table S1). Further, flutamide is used as an anti-androgen in hormone treatment of prostate cancer, and the activity of flutamide was synergized by simultaneous treatment with Resv [21].

Drugs used for metabolic disorders are modulated by Resv as well: The effects of simvastatin (cholesterol-reducing statin) and metformin (reducing hepatic gluconeogenesis) in combination with hydroxymethyl-butyrate (leucine metabolite) were potentiated by simultaneous Resv treatment [22]. The increased relaxation of vascular smooth muscle by hydralazine in combination with Resv is an additional example [23] (Table S1).

The above described combinatory effects of Resv with various drugs *in vitro* are supported by *in vivo* experiments [24,25], as shown in Supplementary Table S2. Together with Resv, piceatannol (a tetrahydroxy-stilbene), when used at 20 mg/kg/day, injected intraperitoneally (i.p.) five days/week for 18 days, potentiates the anti-tumor effect of cisplatin [26].

The necessary dose of metformin was reduced by simultaneous treatment with Resv (about 12.5 mg/kg body weight (b.w.)/day) and hydroxymethyl-butyrate [22], which will reduce the side effects of metformin. On the other hand, Resv (about 11 mg/kg b.w./day) does not add anything to the anti-atherogenic effect of atorvastatin [27] or the co-treatment of phenelzine and Resv to increase the glucose tolerance in mice fed a high fat diet [28].

These steps towards the suggested medical endpoint are promising in most cases as they may reduce the doses needed for a specific medical condition, which will reduce the levels of side effects. This has been addressed in a few experiments where ototoxicity because of cisplatin treatment was prevented by 10 mg Resv/kg taken i.p. for five days [29] or where 10 mg Resv/kg b.w./day per orally (p.o.) diminished doxorubicin-induced losses of body and heart weight [30].

### 2.2. Combinatory Effect—Do the Dietary Components Work in Cooperation?

As described above, a significant biological effect of Resv, as part of the daily diet, is only possible if one suggests a combinatory effect with other components of the diet. Have such effects been documented? In many published experiments, synergistic effects have been shown *in vitro* with combinations of Resv and other naturally occurring compounds (Table S3). Synergism is claimed in several cases, but not documented. The combination of Resv with various stilbenes did show synergism (with pterostilbene and polydatin) when analyzing the antioxidant effects as well as the cytostatic effects [31,32]. The combination of curcumin with Resv has been tested in various *in vitro* models, such as antioxidant capacity, cytostatic effect, and induction of apoptosis [33] (Table S3). Various flavonoids have been tested in combination with Resv, including chrysin, quercetin, catechin, genistein, and combinations of several flavonoids. The effects varied depending on the measured activity and combinations of compounds tested. In general, the same response, but at lower concentrations, was most often observed by using the combined compounds, e.g., combined exposure of HeLa cells to genistein and Resv [34]. The combination of Resv with non-polyphenols also showed interesting combinatory effects *in vitro*: ω-3 fatty acids, docosahexaenoic acid (DHA), and eicosapentaenoic acid (EPA), caused a stronger anti-inflammatory response in combination with Resv [35]. *In vitro* studies of a combination of Resv with other naturally occurring compounds also analyze anti-amyloid activity [36], mitochondrial function [37], nitric oxide induction [38], triglyceride accumulation [39], fatty acid oxidation, and AMPK and Sirt1 activity [40,41].

Vitamin E and C (with lipophilic and hydrophilic antioxidants, respectively) have been tested in combination with Resv in relation to cardio- and neuro-protection. A stronger response was observed by their combination relative to the single compounds (2.5 mg Resv/kg b.w. for 15 days and/or 0.3 mg/kg γ-tocotrienol for 30 days relative to ischemia/reperfusion or lindane-induce neurotoxicity in mice was partly counteracted by a mixture containing 50 mg vitamin E/kg, 50 mg vitamin C/kg, 20 mg α-lipoic acid/kg and 5 mg Resv/kg b.w.) but the dose-dependent response was not fully elucidated [42,43]. Lipoic acid (an organosulfur compound) was also tested alone and with Resv, and Saleh *et al.* showed a similar neuroprotective effect with the combination of lipoic acid and Resv (2 × 10^−5^–2 × 10^−6^ mg/kg Resv in combination with 0.005 mg/kg lipoic acid) to reduce the infarct volume to that found with a 100× higher level of Resv alone [44]. With the aim of reducing carcinogenesis in experimental animals, several combinations with Resv have been published, including ellagic acid, curcumin, and ursolic acid, all of which are polyphenols. Despite the fact that different animal models and biomarkers are used, in general the combined effect is stronger than the effects of the single compounds, but it is not clear whether a synergistic effect is obtained [45,46,47]. Other polyphenols and other naturally occurring compounds have been tested in combination with Resv in relation to chemopreventive potential as well (Table S4).

The combined effects on metabolic disease parameters have also been investigated. A combination of hydroxymethyl-butyrate (2 g/kg diet) and leucine (24 g/kg diet) with Resv (12.5 mg/kg diet) showed a strong combinatory effect, in the form of increased insulin sensitivity and improved inflammatory stress biomarkers [40], likely due to mechanisms similar to the combination of Resv (200 mg/kg b.w. by gavage) with rapamycin (1.5 mg/kg b.w. by gavage, inhibition of mTOR signaling cascade) [48].

A few clinical trials with combinations including Resv have been performed (Table S4) focusing on the effect of Resv (20 mg/day) and calcium fructoborate (112 mg/day) on high-sensitive CRP and angina episodes [49] and the combinatory effect of Resv (120 mg/day) and quercetin (225 mg/day) for six days on exercise-induced lipid peroxidation [50]. In both trials, a significant effect was observed for combination treatments.

Based on the identified papers, the combinatory effects of Resv and other naturally occurring compounds are shown in many cases, but experiments are often not showing synergism mainly because the experimental designs did not allow for drawing such conclusions. Enhanced responses are obtained for Resv in combination with compounds that are structurally and/or functionally Resv-like.

### 2.3. Complex Mixtures—Effect of Resveratrol

In contrast to the experiments with combinations of two components where synergism may be determined by calculation of the combination index, as described by Chou [7], other types of compound interactions may be determined by multivariate analysis of the biological effects of mixtures containing many bioactive compounds. Such types of analysis have not yet been done with extracts containing Resv.

Experiments with various Resv-containing mixtures are shown in Table S5. A few *in vitro* experiments show the effects of mixtures exposed to cells. Resv, in combination with epicatechin, cyanidin, and quercetin or grape extract (GE), showed greater anti-apoptotic and antioxidant effects in comparison to single compounds [51], while other combinations showed effects on cell migration [52]. Different extracts have been tested in various animal models; in only a single case was the biological activity studied on sub-parts of the mixtures, e.g., single components, together with the complete mixture: grape seed extract (200 mg/kg food) and a Concord grape juice extract (183 mg total polyphenol/kg food) were tested in combination with Resv (400 mg/kg food) *vs.* the three parts alone for the effect on amyloid-β (Aβ)-mediated neuropathology and cognitive impairments in a mouse model [53]. Combination treatment resulted in better protection against cognitive impairments compared with the single sub-parts [53].

In total, four human trials were identified where Resv-containing mixtures were applied. Qureshi *et al.* showed that Resv in a mixture also containing pterostilbene, morin hydrate, quercetin, δ-tocotrienol, and riboflavin (25 mg Resv out of 150 mg bioactive components) reduced nitric oxide in serum and various inflammation markers in elderly people [54], and a mixture of Resv, quercetin, and δ-tocotrienol reduced the serum levels of nitric oxide, and CRP, γ-GT activity, and blood pressure [55] (Table S5).

In general, Resv mixtures show promising results in both animal and human studies, but better quality studies are needed as measurement of the contribution of the various components of the mixture is often lacking. Having different preparations with varying contents of the components makes multivariate analysis possible. Otherwise, we still only know the combination effect of the specific mixture under investigation.

### 2.4. How to Get Further?

One of the challenges for researchers in the field of Resv (and many other bioactive components of the human diet) is that the compound does not have one specific cellular target, but interferes with several (many?) targets at the same time, with relatively low affinity. This is in contrast to most of the designed drugs, which are supposed to have one target that they bind to with high affinity. The aim in drug discovery is for specificity of drugs acting on specific targets, whereas compounds like Resv have various activities. Therefore, the combined biological effects of Resv and other naturally occurring bioactive compounds are of specific relevance when discussing the health effects of Resv in the human diet. Several combined health effects have been identified in the animal and clinical studies described above.

These combined biological effects of Resv and other dietary compounds cannot be predicted based on the biological activities observed for Resv and other compounds alone [56]. Because of this lack of predictability, the number of combinations that has to be tested is enormous. A new strategy to identify new and relevant interactions of Resv with drugs (drug–drug interaction) is described by Mahida *et al.* [57] and may be translated to screening of combined health responses to Resv-containing mixtures (e.g., the diet).

Testing the combinatory effects has to include statistical analysis for combinatory effect [7] to classify the description of the combined responses as either synergistic, additive, or antagonistic. New mathematical and statistical tools for analyzing multi-component mixtures have to be developed. Focusing on the biological effects of complex mixtures, e.g., the diet, better study designs should be used in the future—designs that permit precise analysis of the levels of the bioactive components. Multivariate analysis may point out the most relevant players of the mixture as well as the interaction of the compounds found in the mixture.

## 3. *In Vivo* Exposure to Resveratrol: What Is the Relevant Dose and What Is the Actual Dose?

To predict the biological effects of low doses (as part of the diet) or high doses of Resv with a therapeutic intention, the actual levels of Resv in the body need to be known. As will be seen below, the measurement of the level of Resv in the water-based part of the body only may underestimate the level of Resv.

Because of the overwhelming data from rodents, the present focus is on humans and pigs are used as a model organism. The pig is a relevant model for humans because it is close in physiology to humans so there is the possibility of similar drug dosing and pharmacokinetics [58]. Given the low bioavailability of Resv and its metabolites, similar to other naturally occurring compounds, it is a challenge to determine the relevant dose. A full recovery of the compound and its metabolites is needed in the bloodstream and the relevant organs.

The low bioavailability of Resv is observed as low levels of the compound in the bloodstream. Experiments conducted in pigs or humans showed that the peak plasma level for Resv (pigs: 5.9 mg/kg intake: 0.23 µM in plasma, human: 25 mg/70 kg intake: ~2 µM in plasma) is found after 30 min [5,59,60]. In the literature, plasma concentrations are given as average concentration (C_av_), mean compound concentrations throughout the time of measurements, and maximum concentration (C_max_)—the compound concentration that reaches its maximum level in plasma after uptake. During administration of 0.5–5.0 g of Resv/day to 40 healthy subjects for 29 days, C_av_ and C_max_ for the parent Resv ranged from 0.04 to 0.55 µM and 0.19 to 4.24 µM, respectively [61]. Additionally, the glucuronide- and sulfate-conjugated metabolites were found to a higher extent compared to parent Resv with the C_av_ and C_max_ values for Resv-3-*O*-sulfate ~10 times, for Resv-3-*O*-glucuronide, ~6 times, and for Resv-4′-*O*-glucuronide ~5 times and ~2.5 times higher, respectively. A pharmacokinetic study in dogs showed that sulfate and glucuronide metabolites of Resv were found to a higher extent than the Resv in the plasma upon oral Resv treatment for 13 weeks [62]. The results were comparable (relative levels of metabolites) with the human data, but suggested higher conjugation of Resv in humans. Administration of Resv to humans at doses of 0.5 and 1 g per day for eight days resulted in no parent Resv detection; nevertheless, up to five different metabolites could be determined in the plasma of the patients, and the dominating metabolite was Resv sulfate glucuronide. The level of the metabolite nearly doubled (from 13 to 22 µM) by increasing the dose from 0.5 to 1.0 g [63]. However, simultaneously, both Resv and its metabolites could be quantified in colorectal tissue samples (both normal and tumor tissue).

The pharmacokinetics may dramatically change depending on the food matrix in which Resv is found. After a single dose of red wine or GE, no free Resv was observed in either the plasma or urinary excretion samples when Resv and piceid were part of the matrix [64], but both *cis-* and *trans*-piceid were observed in plasma and urine. Only glucuronide conjugates were observed in the plasma, but both sulfate and glucoronate conjugates were collected in the excreted samples. The reason for the absence of free Resv isomers in the samples was probably the low amount of Resv in both red wine (16.8 mg/L) and GE (11.8 mg/L) compared to the exposure levels in other studies.

When Resv is part of wine or other dietary components, it results in higher uptake than the pure compound. Mean Resv concentration in wine is indicated as ~8.2 µM [65], with large variation. Indeed, a 1 µM mean increase in plasma Resv level was observed after administration of Resv in combination with 300 mL of white or red wine for 15 days [66]. As part of this, clearance of Resv from the body is delayed when Resv is consumed in a dietary matrix [64,67]. On the other hand, Vitaglione *et al.* reported that Resv or Resv metabolites were not detected after consumption of wine containing ~3.6 µM Resv in the serum of six out of 10 healthy test subjects [68]. La Porte *et al.* reported that maximal Resv concentration in the plasma after 2 g supplementation twice/day for seven days and area under the curve, as an indicator of the total plasma concentration throughout the measurement, was significantly higher in subjects fed a standard breakfast compared to a high fat breakfast [69]. Neither quercetin supplement nor 5% alcohol showed significant difference in pharmacokinetics parameters [69]. On the other hand, alcohol or other components of wine are of no importance for Resv metabolism as there was no difference in phase II, glucoside, and gut microbial metabolite profiles after four weeks of intervention with red wine or dealcoholized red wine [70]. Comparison of acute (4 h) and chronic (15 days) administration of a GE beverage containing 1.1 mg stilbene showed similar levels of metabolites in the samples for 4 h acute and chronic exposure [71] indicating that GE treatment by itself does not change the metabolism.

The metabolism of Resv by human microbiota is another confusing factor in relation to the level of Resv and metabolites in the body. A changed metabolite profile was observed when using the human fecal microbiota [72], which is why the metabolism by gastrointestinal microbiota is also relevant.

Tissue concentrations of Resv and metabolites upon uptake have been poorly described. In colorectal tissue, oral intake of 0.5–1 g Resv per day for eight days resulted in ~20–675 nmol Resv/g tissue, whereas a 0.5 g dose resulted in 86 nmol Resv-3-*O*-glucuronide/g, which was not further increased by a 1 g dose [63]. In rats, ~80% of the total radioactivity was recovered 2 h after the administration of 50 mg [^3^H]-Resv/kg b.w., with ~76% in gastrointestinal tract, ~1.7% in plasma, and ~1.7% in tissues [73]. At 18 h after administration, 6% of radioactivity was recovered in the gastrointestinal tract, plasma, and tissues, and ~5% was recovered after excretion in urine and feces, suggesting that nearly 90% of the dose administered has been absorbed. Lou *et al.* analyzed the distribution of Resv and its metabolites in rats after intravenous administration of Resv at 20 mg·Resv/kg b.w. [74]. Total Resv and metabolite concentration over time was found to have the highest concentration in the kidneys, followed by the plasma, heart, liver, and brain. Unmodified Resv was most frequent in heart tissue 0.5 h after administration. Sulfated metabolite was more pronounced in the urine, liver, and brain, whereas Resv-3-*O*-glucuronide was found at higher levels in the plasma and kidney compared to other tissues. In pigs, nearly 74.5% (parent Resv and 18 metabolites) of the 5.9 mg/kg b.w. Resv administered intragastrically was recovered after 6 h in the gastrointestinal tract (65.1%), urine (7.7%), bile (2.1%), and various organs (0.5%) [75]. Cai *et al.* reported that one-week oral administration of either 5 mg (dietary-relevant level) or 1 g of [^14^C]-Resv per day led to quantification of parent Resv and metabolites in the colorectal tissue even at significant dietary concentrations (C_max_ was 0.6 µM for 5 mg and 137 µM for a 1 g dose) [76].

Resv is not well soluble in water [77,78,79] and binds to plasma proteins, lipids, and lipoproteins [80,81], which is why these fractions should be considered. Also, human plasma levels of parent Resv are low compared to the dose administrated. Walle *et al.* reported that ~70% Resv was absorbed after oral administration with [^14^C]-labeled Resv [5]. After oral administration, Resv is mainly absorbed by diffusion through the epithelia and quickly metabolized in the liver and intestines, which to a large extent explains the low levels of the parent compound in the plasma.

The abovementioned studies show that resveratrol is easily taken up and quickly metabolized when given orally; several factors change the bioavailability. The metabolism of other resveratrol derivatives is not reviewed here but may give a higher bioavailability. Also, the dietary composition and the gastrointestinal microbiota may change the metabolism and absorption and may explain the variation in metabolism observed among humans. The fact that only 70%–90% of the administrated resveratrol can be recovered may indicate that some amount of Resv, because of its lipophilicity, is still found in the tissue. This was observed in human colonic tissue at low doses of Resv (5 mg/day). More studies on bioavailability are needed in humans, especially focusing on the modulating effects of the diet on the uptake and metabolism of Resv; however, the use of the pig as a model organism should be continued in additional pharmacokinetic studies as these data would be more valuable compared with data from rats and mice.

### How to Increase and Target the Bioavailability?

The value of the tremendous amount of *in vitro* studies concerning the effects of Resv on various diseases is hampered by its low bioavailability. An increased bioavailability of Resv is of special relevance when the therapeutic effect of Resv is in question (using high doses) or when specific tissues should be targeted. Different interventions through several uptake routes have been performed in order to increase the absorption, bioavailability, and efficacy of Resv. Most of the studies use rats, whereas only a few tested the effects in humans. The safety, pharmacokinetics, and pharmacodynamics of a micronized Resv formulation called SRT501 were the focus of a clinical study in which 5 g/day was administered for around 14 days to colon cancer patients with hepatic metastasis [82]. The hypothesis behind the study was that because Resv is poorly soluble in water, decreasing the particle size might improve the absorption. After ingestion of SRT501, the mean plasma maximum concentration (C_max_) for Resv was found to be 1.9 ± 1.4 ng/mL (~8.5 µM). When compared with previous studies from Boocock *et al.* [83] and Brown *et al.* [61], micronized Resv showed ~2–3.5-fold higher mean plasma concentration. A second application to enhance the bioavailability is lipid-core nanocapsulation of Resv. Nanocapsulation of Resv not only improved the distribution of Resv in the brain, liver, and kidneys in rats, but also diminished gastrointestinal damage relative to free Resv [84]. Administration of solid lipid nanoparticles containing Resv (5 mg/kg) via the intraperitoneal route also enhanced the delivery of Resv to the brain ~6-fold in rats compared to free Resv, but distribution to other organs was unchanged or decreased [85]. Further, oral administration of 15 mg/kg zein-based Resv-loaded nanoparticles to rats lessened the endotoxic shock induced by LPS [86]. The plasma C_max_ values for oral zein-based nanoparticle treatment were also found to be significantly higher (~1.7 µM) than Resv administered in a solution (~0.87 µM). Resv-loaded Eudragit RL 100 nanoparticles also showed higher plasma levels in rats compared to pure Resv treatment, as well as longer release time [87]. The soluble galenic form (soluble lipid formulation containing polysorbate 20 and polyglyceryl-3-dioleate) of Resv improved plasma C_max_ Resv levels (5.7 µM) in healthy humans compared to the administration of Resv in powder form (470 nM) [88]. Also, metabolite levels were found to be higher in the soluble administered group. The same study indicated that, compared to the powder form, the soluble form showed higher anti-inflammatory activity in mice fed a high fat diet.

In order to increase the transmucosal bioavailability and plasma levels of Resv, a lozenge formulation has been developed recently [89]. Around 150 mg of Resv was administered to two healthy subjects and the maximum blood plasma level was found to be around 323 ± 2.12 ng/mL (~1.4 µM) 15 min after administration, which indicates higher absorption compared to the studies done with higher doses using gastrointestinal uptake. Oral administration of Resv coated with *N*-trimethyl chitosan-*g*-palmitic acid surface-modified solid lipid nanoparticles resulted in significantly higher plasma concentrations in mice compared to Resv administrated in suspension counterparts [90]. Oral administration of 50 mg/kg Resv-loaded carboxymethyl nanoparticles to rats enhanced the relative bioavailability of Resv compared to Resv only [91]. In a recent study, Scalia *et al.* [92] observed that coating the lipid microparticles loaded with Resv with chitosan significantly increases the *in vivo* permeation of Resv into the human stratum corneum.

New formulations may be one way to enhance the bioavailability and target Resv to specific organs. The enhanced bioavailability may change the toxicity profile, which is why the toxicity of the formulated Resv must be analyzed together with the proposed health-promoting effect of the new Resv formulation.

## 4. Preventive *vs.* Therapeutic Effects of Resveratrol

To date, only a few clinical studies have been published analyzing the health-promoting effects of Resv, with a low number of subjects. In addition, those studies have only analyzed the therapeutic effects of Resv; no preventive human studies have been published yet. The basic reason for this is the impossibility of doing long-term (5–10 years or life-long) exposure clinical trials on Resv. Monitoring the subjects for that long a time, as well as funding this kind of research, is nearly impossible. A few studies have been conducted to prevent prostate cancer, with a low success rate [93]; based on the exposure time and concentrations of compounds, one has to ask whether these are preventative or therapeutic trials. If such preventative clinical trials are not performed in humans, we will not discover whether Resv has disease-preventing activity or not. Further, such information is also requested by authorities like the FDA (Food and Drug Administration) and EFSA (European Food Safety Authority) to support health claims. The designs of trials to show the preventative effects of Resv are different from therapeutic effect trials. As pointed out by Smoliga *et al.*, “trials targeting healthy humans are often fundamentally flawed owing to inappropriate use of paradigms only applicable to populations with overt clinical disease and the consequent misleading (typically negative) results can severely retard advancement of drug development” [94].

Given the challenges to getting the “right” human data, we suggest that such long-term analyses can be done in species taxonomically and physiologically close to humans. Many preventive cancer studies have been performed on experimental animals, especially in rodents, but only a small number of studies in other, more relevant animals such as pigs have focused on the preventive responses of Resv.

In various animal models, a number of preventive studies have been conducted. Focusing on the available data from pigs, Resv showed cardioprotective effects in experimental setups such as diet-induced metabolic syndrome models, one of the high risk factors of cardiovascular death. Previously, Resv administration was shown to have preventive effects on risk factors for cardiovascular health in Yorkshire swine with metabolic syndrome [95,96]. Another example showed that Resv (100 mg/kg/day) improved regional left ventricular function and restored perfusion when Resv was added together with a hypercholesterolemic diet for four weeks of pretreatment followed by seven weeks of induced ischemia (11 weeks Resv treatment in total) [97]. Further, Resv caused lower body mass index, insulin, total cholesterol, LDL, and systolic blood pressure by using the same design [98]. Additionally, experiments on non-human primates showed promising results. Eighteen months of Resv treatment (200 mg/kg/day) improved several cognitive abilities, such as spontaneous locomotor activity, working memory, and spatial motor performance, in gray mouse lemurs [99]. Another study with the same dose of Resv for 33 months improved insulin sensitivity in gray mouse lemurs [100]. A 24-month administration of Resv to Rhesus monkeys (first year: 80 mg/day; second year: 480 mg/day) prevented β-cell differentiation and morphological changes in the pancreatic islets [101], and arterial wall inflammation and stiffening induced by a high calorie diet [102].

As mentioned above, Resv effects are tested in animal models in preventive designs, whereas clinical studies mainly use therapeutic designs to test the properties of Resv. Experiments done in animal models, mostly rodents, provide promising results about the potential of Resv in disease prevention [103]. Focusing on the clinical data on Resv published from 2010 until now, some conclusions may be drawn.

### 4.1. Studies Including Healthy Subjects

Exposing healthy subjects to Resv is similar to a preventive design, however, it is possible that the disease development may have already been initiated at the cellular level but is still not visible to the investigating physician. In female subjects, Yoshino *et al.* reported that 75 mg/day Resv supplementation for 12 weeks did not alter variables such as insulin sensitivity, resting metabolic rate, inflammatory markers, or body composition [104] (Table 1). Further, no expressional change was observed for molecular markers including SIRT1, NAMPT, and PPARGC1A, or for activity of AMPK. In older adults, memory performance was improved by 200 mg/day after 26 weeks of Resv intervention but the glucose, insulin, inflammatory marker levels, and lipid profile were unchanged compared to the placebo group [105]. Another study suggested that four weeks of 400 mg/day Resv might be protective against atherosclerosis [106] (Table 1).

### 4.2. Studies Including Subjects with Metabolic Challenges

In 2011, Timmers *et al.* observed that Resv (150 mg/day for 30 days) had the same effects as caloric restriction in obese humans, such as improved metabolic function, lowered energy expenditure, improved insulin sensitivity, and lowered inflammation markers [107] (Table 2). In type 2 diabetic (T2D) patients, four weeks of 2 × 5 mg oral Resv administration resulted in decreased insulin resistance, but did not alter the β-cell function [108]. In general, the doses of Resv used varied from 150 mg/day to 2 g/day and the length of the intervention varied from four weeks to three months (Table 2), with an accumulated Resv dose ranging from 4.5 g [107] to 135 g [109]. There is no clear dose- or time-dependent effect of Resv in these clinical trials, but varying relevant parameters are modulated differently in the different clinical trials. In a clinical study, patients with either diabetes mellitus or hypercholesterolemia were administered GE or GE containing 8 mg of Resv for six months [110]. Resv intervention significantly reduced apolipoprotein-B and oxidized low-density lipoprotein (LDL) levels, but did not alter blood glucose levels or renal, hepatic, and thyroid function (Table 2).

### 4.3. Studies Including Subjects with Cardiovascular Challenges

In a clinical trial with one-year follow up, subjects with high risk of cardiovascular disease were administered either Resv, GE enriched with Resv (8 mg), or conventional GE that does not contain measureable amounts of Resv for six months and then a doubled dose for the next six months [111]. Resv-treated persons showed significant improvement in inflammatory and fibrinolytic status, suggesting the preventive effects of Resv in cardiovascular disease. On the other hand, there was no difference in glucose, LDL, or high-density lipoprotein (HDL) levels between the groups administered GE or GE containing Resv, and no clear dose-response effect was observed. Further, in T2D hypertensive patients with coronary artery disease, 12 months of receiving GE containing Resv (8 mg) did not alter the blood pressure, glucose, or inflammatory markers except for a significant decline in alkaline phosphatase and Interleukin-6 (IL-6) [112]. Improved left ventricular diastolic function and endothelial function, as well as a decrease in platelet aggregation and LDL levels, were observed in patients with coronary artery disease after 10 mg/day Resv intake for three months [113]. A dose of 150 mg/day Resv for four weeks did not change the levels of metabolic risk markers related to cardiovascular fitness (HDL cholesterol, LDL cholesterol, insulin, glucose, triacylglycerol, and systolic blood pressure) but increased diastolic blood pressure and heart rate in slightly obese subjects compared to the placebo treatment [114]. Table 3 gives more details and compares different studies analyzing the cardiovascular parameters in response to Resv treatment.

### 4.4. Studies Including Subjects with Cancer

A few studies have investigated the possible chemopreventive potential of Resv. Nguyen *et al.* demonstrated that two weeks of administration of 80 mg of grape powder containing Resv (0.073 mg), but not Resv tablets (Resv content: 3.9 and 15.5 mg in 20 mg and 80 mg tablets, respectively), for did not inhibit the Wnt pathway, which is an important pathway in colon cancer development, in cancerous tissue but significantly inhibited the pathway in normal colonic mucosa [115], suggesting a chemopreventive role of resveratrol. In colorectal cancer patients with hepatic metastasis, Resv treatment (5 g/day, mean exposure for 14 days) enhanced the level of cleaved caspase-3 in hepatic tissue, suggesting higher apoptotic activity in cancerous tissue compared to placebo-treated counterparts [82]. On the other hand, another study used the same Resv formulation at the same dose in patients with relapsed and or refractory multiple myeloma, and observed renal toxicity in five out of 24 patients [116]. Renal failure is one of the specific clinical features of multiple myeloma and can be observed in nearly half of the patients throughout the disease [117]. Resv treatment cannot be pinpointed as the reason for renal impairment in those patients. In a recent study, the levels of cytoprotective enzyme NQO1 and protein carbonyl concentrations were found to be higher in colorectal mucosa tissues in patients receiving 5 mg Resv for 14 days compared to their control counterparts [76] (Table 4).

### 4.5. Studies Including Subjects with Alzheimers

A 52-week dose-escalation study (beginning with 500 mg/day and ending with 2 × 1 g/day) investigated the effect of Resv in Alzheimer’s disease patients. It was observed that Resv increased the brain volume loss [118]. Additionally, the levels of Aβ40 in cerebrospinal fluid and plasma were reduced more in placebo group patients than in the Resv-treated group.

## 5. Concluding Remarks

The challenges for the research on Resv that we raise here focus on the proposed combinatory effect of Resv with other dietary compounds (and drugs), the assumed low bioavailability, and the difficulty of proving preventive activity in human tests.

The identified clinical studies investigated the therapeutic effects of Resv, except for four studies in healthy subjects [104,105,106,119] and the study by Tome-Carneiro *et al.* [111], where the study subjects had a high risk of cardiovascular disease. It is likely that the “disorders” were already initiated in many of the subjects before the start of Resv treatment but were not visible yet, which is why these are not clear preventive studies. Considering the various studies shown above, the effect of Resv is dependent on the condition of the subject. The doses used differ significantly, and the number of persons included is still too small to verify a dose-dependent effect. In some cases, even small levels of Resv gave a response where high doses did not. Including knowledge from animal studies, a direct translation of the preventive effects observed here to humans is not possible. However, it is safe to say that Resv prevents the development of coronary vascular disease, improves insulin sensitivity, reduces serum glucose, and prevents the development of cancers of the skin, colon, and prostate in animal models. The same data in humans are limited or non-existent.

A reliable preventive effect in humans is founded on the combined effect of Resv and the other bioactive components present in the human diet. Here, “synergism” is a very much-used term, but in many cases, “synergism” is postulated without testing it. Testing the activities of complex mixtures in animals and humans should be founded on a precise description of the contents of components in the mixture.

The low bioavailability of Resv, which is most obvious in therapeutic experiments, could be enhanced by new formulations and/or routes of administration.

To advance our current knowledge about the preventive activity of Resv, which has mainly been gained from rodents, we suggest studies with a preventive design in humans or preparing further preventive tests in pigs and non-human primates.

For preventive studies in humans, treatment with Resv should be long-term (5–10 years) in order to see a preventive effect, and levels of Resv should be not too far from the dietary exposure level of 2–5 mg/day. To go much higher in terms of Resv doses, long-term safety experiments have to be done in advance. Further, recent data did show that measurable amounts of resveratrol are observed in colonic tissue even up to seven days after intake of 1 × 5 mg Resv [76]. We further suggest that the quality of clinical trials in future should be improved through an increased number of participants, appropriate study designs, and biomarkers relevant to healthy participants [94]; otherwise, the preventive and therapeutic properties of Resv in humans will remain as only a hypothesis because preventive and therapeutic effects in humans are only supported by *in vitro* and *in vivo* model organism studies.

## Figures and Tables

**Table 1:** Effect of resveratrol treatment on parameters in healthy subjects. Changes in parameters are shown relative to baseline measurements. “↑”, “↓” and “-” indicate induction, reduction, and no effect, respectively. The comparisons are between the placebo and intervention group, unless indicated with asterisks.

Participant Condition (Design of the Study)	Parameter	Parameter Change		Dose	Duration	Reference
**Heathy young adult** (Double-blinded/Placebo-controlled/crossover) *N* = 22	Total hemoglobin	↑		250 and 500 mg	2 separate days (7 ± 2 days between)	Kennedy *et al.*, 2010 [119]
	Oxygenated hemoglobin	-				
	Deoxygenated hemoglobin	↑				
	Task related differences	-				
	Cognitive task performance and mental fatigue	-				
**Healthy non-obese with healthy glucose tolerance** (Randomized/Double-blinded/ Placebo-controlled) *N* = 29	BMI, Free-fat index, Free fat, Subcutaneous abdominal fat volume, intra-abdominal fat volume, intra-hepatic triglyceride content	-		75 mg/day	12 weeks	Yoshino *et al.*, 2012 [104]
	Glucose, Insulin, HOMA-IR	-				
	Free fatty acids, Total cholesterol, LDL-c, HDL-c, Triglyceride	-				
	Leptin	-				
	Adiponectin, IL-6, CRP	-				
	Resting metabolic rate	-				
	Hepatic insulin sensitivity rate	-				
	Systolic and Diastolic blood pressure	-				
	White blood, Red blood cell count, Platelet	-				
	Hemoglobin, Hematrocit, MCV, MCH, MCHC	-				
	Blood urea nitrogen, Total protein, Albumin	-				
	AST, ALT, Alkaline phosphatase, Bilirubin	-				
	Muscle or adipose tissue gene expression for					
	*SIRT1*	-				
	*NAMPT*	-				
	*PPARGC1A*	-				
	*UCP3*	-				
**Healthy older adults** (Double-blinded/Placebo-controlled) *N* = 46	Memory retention	↑		200 mg/day	26 weeks	Witte *et al.*, 2014 [105]
	HbA1c	↓				
	Insulin	↑				
	Total cholesterol	↑				
	Leptin	↑				
	BDNF	-				
	LDL:HDL ratio, Triacylglycerides	-				
	IGF-1	-				
	TNF-α	↓				
	IL-6, hsCRP	↓				
	Body fat, BMI	-				
	Systolic blood pressure	-				
	Diastolic blood pressure	↓				
**Subjects with normal heart rate and Blood pressure** (Randomized/Triple-blinded/Placebo-controlled) *N* = 44		Resveratrol	Placebo	400 mg Resv + 400 mg grape skin extract + 100 mg quercetin	30 days	Agarwal *et al.*, 2013 [106]
	Insulin	↓	-			
	Glucose	-	-			
	INF-γ	↓	-			
	IL-1β	-	↓			
	IL-6, TNF-α	-	-			
	Leptin	-	-			
	ICAM, VCAM	↓	-			
	IL-8	↓	-			

Abbreviations: ALT, alanine aminotransferase; AST, aspartate aminotransferase; BDNF, brain-derived neurotrophic factor; BMI, body mass index; CRP, C-reactive protein; HbA1c, glycated hemoglobin; HDL-c, high-density lipoprotein-cholesterol; HOMA-IR, homeostatic model assessment-Insulin resistance; hsCRP, high-sensitivity C-reactive protein; ICAM, intercellular adhesion molecule; IGF-1, insulin-like growth factor 1; IL, interleukin; INF-γ, interferon gamma; LDL-c, low-density lipoprotein-cholesterol; MCHC, mean corpuscular hemoglobin concentration; MCV, mean cell volume; NAMPT, nicotinamide phosphoribosyltransferase; PPARGC1A, peroxisome proliferator-activated receptor gamma coactivator 1-alpha; SIRT1, Sirtuin 1; TNF-α, tumor necrosis factor alpha; UCP3, mitochondrial uncoupling protein 3; VCAM, vascular cell adhesion molecule.

**Table 2:** Effect of resveratrol in subjects with metabolic challenges. “↑”, “↓” and “-” indicate induction, reduction, and no effect, respectively. The comparisons are between the placebo and intervention group, unless indicated with asterisks.

Condition of the Subject (Design of the Study)	Parameter	Parameter Change	Dose	Duration	Reference
**Healthy obese subjects** (Randomized/double-blinded/ /crossover design with 4-weeks washout) *N* = 11	Glucose	↓	150 mg/day	30 days *	Timmers *et al.*, 2011 [107]
	Insulin	↓			
	HOMA index	↓			
	Triglycerides	↓			
	Leptin	↓			
	TNF-α	↓			
	Leukocytes	↓			
	ALT	↓			
	Non-esterified fatty acids	-			
	Adiponectin, CRP, IL-1β, IL-6, IL-8	-			
	Hemoglobin, Erythrocytes, Thrombocytes	-			
	Leukocytes	-			
	Urea, Creatinine AST, Bilirubin, Total protein, Albumin	-			
**Healthy obese subjects** (Randomized/double-blinded/placebo-controlled/parallel-group trial) *N* = 24	Glucose, Insulin, HOMA index, HbA1c	-	3 × 500 mg/day	4 weeks *	Poulsen *et al.*, 2013 [120]
	Cholesterol, HDL, LDL, Triglycerides	-			
	Leptin,	-			
	hsCRP, IL-6, TNF-α, MCP-1	-			
	Leukocytes, ALT	-			
**Obese with mild/ moderate hyperglycemia** (Randomized/double-blinded/crossover trial) *N* = 8	Glucose, Insulin, HOMA-IR	-	1 g (week 1) 2 g (week 2)	2 weeks *	Dash *et al.*, 2013 [121]
	Triglycerides	-			
	ApoB-48	↓			
	Catabolic rate	-			
	ApoB-100	↓			
	Catabolic rate	↓			
**Type 2 Diabetic patients** (Randomized/Placebo-controlled/double-blinded) *N* = 66	Body weight, BMI	-	2 × 500 mg/day	45 days **	Movahed *et al.*, 2013 [122]
	Systolic blood pressure	↓			
	Diastolic blood pressure	-			
	Fasting glucose, Insulin, HOMA-IR	↓			
	HbA1c	↓			
	HOMA-β	↓			
	Triglyceride, Total cholesterol	-			
	HDL-c	↑			
	LDL-c	-			
	Creatinine	-			
**Type 2 Diabetic patients** (Prospective/open-label/randomized/controlled study) *N* = 62	BMI	-	250 mg/day	3 months *	Bhatt *et al.*, 2012 [123]
	Fasting glucose	↓			
	HbA_1c_	↓			
	Systolic and diastolic blood pressure	↓			
	Total cholesterol, LDL-c, Triglycerides	↓			
	HDL-c	-			
	Urea nitrogen	↓			
	Creatinine	-			
	Total protein	↓			
**Patients with impaired glucose tolerance** (Randomized/open-label/pilot study) *N* = 10	Body weight, Body fat	-	1–2 g/day	4 weeks **	Crandall *et al.*, 2012 [124]
	Cholesterol, HDL-c, LDL-c, Triglycerides	-			
	hsCRP, Adiponectin	-			
	Fasting glucose, Fasting insulin	-			
	HOMA-IR	-			
	Insulin sensitivity (Matsuda index)	-			
	Serum creatinine, AST, ALT	-			
	Systolic and diastolic blood pressure	-			
	Glucose (AUC- 3 h after meal)	↓			
	Insulin (AUC- 3 h after meal)	↓			
**Patients with Metabolic syndrome** (Randomized/double-blinded/placebo-controlled) *N* = 24	Body weight, BMI, Fat mass	↓	3 × 500 mg/day	90 days **	Mendez-Del Villar *et al.*, 2014 [109]
	Waist circumference	↓			
	Systolic and diastolic blood pressure	-			
	Glucose	-			
	Triglycerides, HDL-c, LDL-c	-			
	Glucose (AUC)	-			
	Insulin (AUC)	↓			
	Insulinogenic index	↓			
	Stumvoll index	-			
	Insulin sensitivity (Matsuda index)	-			
**Patients with Non-alcoholic fatty liver disease** (Randomized/double-blinded/placebo-controlled) *N* = 60	Body weight, BMI, Waist circumference, Hip circumference	-	2 × 150 mg	3 months *	Chen *et al.*, 2015 [125]
	Systolic and diastolic blood pressure	-			
	Red blood cell, White blood cell and Platelet	-			
	Blood urea nitrogen, Creatinine	-			
	ALT, AST	↓			
	GGT	-			
	Glucose	↓			
	Insulin	-			
	HOMA-IR	↓			
	C-peptide	-			
	Total cholesterol, HDL-c, LDL-c	↓			
	Triacylglycerol	↓			
	ApoB, ApoA-I	-			
	TNF-α	↓			
	Adiponectin	↑			
	Cytokeratin 18	↓			
	Fibroblast growth factor 21	↓			
**Patients with Non-alcoholic fatty liver disease** (Randomized/double-blinded/placebo-controlled) *N* = 50	Body weight, BMI, Waist circumference, Energy intake	-	500 mg/day	12 weeks ***	Faghihzadeh *et al.*, 2014 [126]
	Hip circumference	-			
	Waist:hip ratio, Metabolic equivalent task	-			
	ALT	↓			
	AST, GGT	-			
	Bilirubin direct, Bilirubin total	-			
	hsCRP, TNF-α, IL-6, Cytokeratin-18, NF-κB	↓			
	Steatosis	↓			

Changes in parameters are indicated as (*) compared to placebo; (**) compared to baseline measurements; and (***) comparison of changes between placebo and intervention group. Abbreviations: ALT, alanine amino transferase; ApoA-I, apolipoprotein A-I; ApoB, apolipoprotein B; AST, aspartate amino transferase; AUC, area under the curve; BMI, body mass index; CRP, c-reactive protein; GGT; gamma-glutamyltransferase; HbA1c, glycated hemoglobin; HDL, high-density lipoprotein; HDL-c, High-density lipoprotein-cholesterol; HOMA, Homeostatic model assessment; HOMA-IR, Homeostatic model assessment-Insulin resistance; HOMA-β, Homeostatic model assessment-beta-cell function; hsCRP, high-sensitivity C-reactive protein; IL, interleukin; LDL, low-density lipoprotein; LDL-c, low-density lipoprotein-cholesterol; MCP-1, monocyte chemoattractant protein-1; NF-κB, Nuclear factor kappa B; TNF-α, tumor necrosis factor alpha.

**Table 3:** Effect of resveratrol treatment on parameters on cardiovascular conditions. “↑”, “↓” and “-” indicate induction, reduction, and no effect, respectively. n.a.: not available. The comparisons are between the placebo and intervention group, unless indicated with asterisks.

Condition of the Subject (Design of the Study)	Parameter	Parameter Change					Dose	Duration	Reference
**Patients treated with primary prevention of cardiovascular disease (3 groups: A: control; B: Grape extract; C: Grape extract + Resv)** (Randomized/Triple-blinded/Placebo-controlled/parallel) *N* = 75		A *vs.* B	A *vs.* C		B *vs.* C		8 mg (6 month)–16 mg (next 6 months)	12 months *	Tome-Carneiro *et al.*, 2012 [111]
	TNF-α, Adiponectin, IL-6,	-	-		-				
	IL-10	-	-		↓				
	IL-6/IL-10, IL-18, hsCRP	-	-		-				
	sICAM-1	-	↓		-				
	PAI-1	-	↓		↓				
**Patients treated with primary prevention of cardiovascular disease (3 groups: A: control; B: Grape extract; C: Grape extract + Resv)** (Randomized/Triple-blinded/Placebo-controlled/parallel) *N* = 75		A *vs.* B	A *vs.* C		B *vs.* C		8 mg	6 months *	Tome-Carneiro *et al.*, 2012 [110]
	Total cholesterol, TGs, HDL-c, Non-HDL-c	-	-		-				
	LDL-c, LDL-ox, LDL-c/ApoB, LDL-c/HDL-c	-	-		-				
	LDL-c/LDL-ox	↑	↑		-				
	ApoB,	-	-		-				
	LDL-ox/ApoB	-	↓		-				
	Non-HDL-c/ApoB	-	-		-				
**Hypertensive patients with coronary artery disease (3 groups: A: control; B: Grape extract; C: Grape extract + Resv)** (Randomized/Triple-blinded/Placebo-controlled/parallel) *N* = 35		A	B		C		8 mg (6 month)–16 mg (next 6 months)	12 months *	Tome-Carneiro *et al.*, 2012 [112]
	Systolic and diastolic blood pressure	-	-		-				
	Total cholesterol, LDL-c, HDL,c, Non-HDL-c, TGs, LDL-c/HDL-c,	-	-		-				
	Glucose	-	-		-				
	HbA1c	-	-		-				
	GGT, ALT, AST	-	-		-				
	ALP	-	↑		↓				
	Creatinine, Albumin, Urate	-	-		-				
	PAI-I	-	-		-				
	Adiponectin	↓	-		-				
	IL-6	-	-		↓				
	hsCRP, TNF-α	-	-		-				
	IL-10, IL6/IL-10	↑	-		-				
**Patients with stable coronary artery disease** (Randomized/Triple-blinded/Placebo-controlled/parallel) *N* = 40		Resv *vs.* baseline		Resv *vs.* placebo			10 mg/day	3 months	Magyar *et al.*, 2012 [113]
	Systolic and diastolic left ventricular function	-		-					
	Diastolic left ventricular function	↑		↑					
	12-h fasting white blood cell count	-		-					
	CRP, TNF-α	-		-					
	Glucose	-		-					
	HbA1c	-		-					
	Total cholesterol, HDL-c, TGs	-		-					
	LDL-c	↑		-					
	Brachial artery FMD	↑		↑					
**Healthy obese adults (Randomized/Double-blinded/Placebo-controlled/cross over)** *N* = 24	Systolic and diastolic blood pressure	-					75 mg/day Resv (ResVida)	12 weeks	Wong *et al.,* 2013 [127]
	Large artery elasticity index	-							
	Small artery elasticity index	-							
	Chronic FMD responses								
	Resting branchial diameter	-							
	Peak branchial diameter	-							
	Chronic FMD	↑							
	Acute FMD responses								
	Resting branchial diameter	-							
	Peak branchial diameter	-							
	Acute FMD	↑							
**Healthy aged physically inactive man** (Randomized/Double-blinded/Placebo-controlled) *N* = 27		Placebo	Resv			Resv *vs.* Placebo	250 mg/day	8 weeks	Gliemann *et al.*, 2013 [128]
	Mean arterial pressure	↓	-			-			
	Heart rate (rest)	↓	-			-			
	Heart rate (max)	-	-			-			
	$V_{O_{2}\text{max}}$	↑	↑			↓			
	${\text{ΔV}}_{O_{2}\text{max}}$	n.a.	n.a.			↓			
	${\text{ΔV}}_{O_{2}\text{max}}{\text{ kg}}^{-1}\;$	↓	↑			↓			
	Glucose	-	-			-			
	Total cholesterol, HDL	-	-			-			
	LDL	↓	-			-			
	Total cholesterol/HDL	↓	-			-			
	TGs	↓	-			-			
	VCAM-1	↓	↓			-			
**Overweight and Slightly Obese Subjects** (Randomized/Placebo-Controlled/Crossover) *N* = 45	Total cholesterol, LDL-c, HDL-c, Total:HDL-c ratio,	-					150 mg/day	4 weeks **	Van der Made *et al.*, 2015 [114]
	Triacylglycerol	-							
	ApoA-I, ApoB-100	-							
	BMI	-							
	Insulin, Glucose, HOMA-IR	-							
	Systolic and diastolic blood pressure	-							
	Heart rate, Mean arterial pressure	-							
	hsCRP, IL-6, TNF-α	-							
	E-selectin, Thrombomodulin, P-selectin	-							
	ICAM-3, sICAM-1, sVCAM-1	-							
**Subjects with non-alcoholic fatty liver disease** (Randomized/Placebo-Controlled/Double-blinded) *N* = 50	ALT	↓					500 mg/day	12 weeks **	Faghihzadeh *et al.*, 2015 [129]
	AST, GGT	-							
	Bilirubin direct, Bilirubin total	-							
	Steatosis	↓							
	TAG, Total cholesterol, LDL-c, HDL	-							
	LDL/HDL, Non-HDL-c	↑							
	Apo-A1	-							
	Glucose, Insulin, HOMA-IR, HOMA-β	-							
	Systolic and diastolic blood pressure	-							
**Overweight/obese individuals with elevated blood pressure** (Randomized/Placebo-Controlled/Double-blinded/Crossover) *N* = 19	FMD	↑					30, 90 and 270 mg or placebo at each weekly visit	4 weeks	Wong *et al.*, 2011 [130]

Changes in parameter are indicated as, (*) comparison of the results after intervention period; (**) comparison of the changes at the end of the study, and without asterisks are comparison between placebo and intervention group at the end of study. Abbreviations: ALP, alkaline phosphatase; ALT, alanine amino transferase; ApoB, apolipoprotein B; AST, aspartate amino transferase; CRP, c-reactive protein; FMD, flow-mediated dilatation; GGT; gamma-glutamyltransferase; HbA1c, glycated hemoglobin; HDL-c, high-density lipoprotein-cholesterol; HDL-ox, oxidized high-density lipoprotein; hsCRP, high-sensitivity C-reactive protein; ICAM-3, intercellular adhesion molecule 3; IL, interleukin; LDL-c, low-density lipoprotein-cholesterol; LDL-ox, oxidized low-density lipoprotein; PAI-I, plasminogen activator inhibitor type 1; sICAM-1, soluble intercellular adhesion molecule 1; sICAM-1, soluble intercellular adhesion molecule type 1; sVCAM-1, soluble vascular cell adhesion molecule 1; TAG, triacylglycerol; TNF-α, tumor necrosis factor alpha; VCAM-1, vascular cell adhesion molecule 1.

**Table 4:** Effect of resveratrol treatment on parameters in colorectal cancer patients. Changes are shown for intervention group relative to a placebo group.

Design of the Study	Parameter	Parameter Change	Dose	Duration	Reference
**Phase I** (Open labelled/Pilot) *N* = 8	Wnt pathway target genes		0.073 mg (80 mg grape extract)	14 days	Nguyen *et al.*, 2009 [115]
	In normal tissue	↓			
	Colon tissue	-			
**Phase I** (Randomized/Double-blinded) *N* = 6	VEGF in serum	-	5 g/day (SRT501)	14 days (mean) (10-21 days)	Howells *et al.*, 2011 [82]
	Prostaglandin E2 in plasma	-			
	IGF-1 in liver	-			
	Cleaved caspase 3	↑			
**Phase I** (Controlled) *N* = 20	AMPK signaling (Tissue from patient)	-	5 mg/day or 1 g/day	6 days	Cai *et al.*, 2015 [76]
	NQO1 (Colorectal mucosa)	↑ (5 mg dose)			
	Protein carbonyl (Colorectal mucosa)	↑ (5 mg dose)			

Abbreviations: AMPK, AMP-activated protein kinase; IGF-1, Insulin-like growth factor-1; NQO1, NAD(P)H dehydrogenase (quinone 1); VEGF, vascular endothelial growth factor.

## Supplementary Materials

The following are available online at http://www.mdpi.com/2072-6643/8/6/353/s1, Table S1: Effects of various drugs *in vitro* modulated by resveratrol (Resv), Table S2: Effects of various drugs *in vivo* modulated by resveratrol (Resv), Table S3: Effects of various naturally occurring compounds *in vitro* modulated by resveratrol (Resv), Table S4: Effects of various naturally occurring compounds *in vivo* in combination with resveratrol (Resv), Table S5: Effects of various mixtures containing resveratrol (Resv).
