# Supplementary material for: Challenges in Analyzing the Biological Effects of Resveratrol
Source: Nutrients. 2016 Jun 9;8(6):353. doi: 10.3390/nu8060353 (PMC4924194; doi:10.3390/nu8060353)
Supplement: Supplementary file 1 [file nutrients-08-00353-s001.zip › nutrients-126957 Suppl Table 01-02 -final.docx]

Supplementary Materials: Challenges in Analyzing the Biological Effects of Resveratrol

Cihan Suleyman Erdogan and Ole Vang

**Table S1.** Effects of various drugs *in vitro* modulated by resveratrol (Resv).

| **Compound** | **Model** | **Effects** | **Referance** |
| --- | --- | --- | --- |
| Vinblastine (Vin) | Cell viability in human 5637 cells measured by MTS activity | Antagonistic effect of Resv (10–100 µM) on the cytotoxic effect of Vin (25 nM).  Using sequential treatment of 5637 cells: Resv at 5 or 10 µM for 48 h first, followed by 48 h treatment with Vin, Resv potentiate the cytotoxicity of Vin. | [1] |
| Paclitaxel (PTX) | Cytotoxicity of MCF-7 cells | Antagonistic effects of the combination of PTX (1.25 nM–40 nM) and Resv (5–160 µM) | [2] |
| Cisplatin (CIP) | Cell viability in human 5637  cells measured by MTS activity | Combinatory effect of Resv (10–100 µM) on the cytotoxic effect of CIP (4 µg/mL) | [1] |
| Cisplatin (CIP) | House Ear Institute-Organ of Corti 1 (HEI-OC1) cell line uses as model. Test for cell viability. | Combination of 20 µM CIP and 0.1 µM Resv is giving less viability than 20 µM CIP alone. | [3] |
| Cisplatin (CIP) or Oxaliplatin (OXA) | Drug resistance in human ovarian A2780 cells | Highest synergy was observed when Resv was administered first followed by the platinum drug (CIP or OXA) 2 h later, and the least synergism was achieved when the compounds together. | [4] |
| Oxaliplatin (OXA) | Cytotoxic effects on colorectal cancer cells, Caco-2 | Resv (50–100 µM) and OXA (1–10 µM) in combination synergistically inhibit cell growth. | [5] |
| Cisplatin (CIP) or carboplatin (CBP) | Effect on post-treatment regrowth of surviving cells | 2 µM CIP, 16 µM CBP or 40 µM Resv do not change the regrowth rate, but only the combination of Resv with either CIP or CBP | [6] |
| Cisplatin (CIP) or Fluorouracil (5-FU) | Cell survival in head and neck cancer-derived tumor-initiating cells sensitive and resistant to chemo-therapeutic compounds | Resv restored the chemo-sensitivity. 100 µM of each compound. | [7] |
| Cisplatin (CIP) or oxaliplatin (OXA) | Cell viability of human ovarian cancer cell lines | A synergistic effect of combination of Resv with CIP or OXA is shown, based on calculations of combination index (CI). The concentrations of the compounds used are not very well described. | [8] |
| Cisplatin (CIP) or Etoposide (ETO) | Cytotoxicity of MCC13 and MCC26 cells was determined using the CCK-8 assay | Based on CI, strong synergy was observed combining Resv and CIP as well as ETO, respectively, in MCC26. In MCC13, the combination of Resv with CIP was additive, but antagonistic with ETO. | [9] |
| Etoposide (ETO) | Cytotoxicity of ETO in HepG2 and HCT-116 cells | Resv (12.5–100 µM) significantly enhanced anti-proliferative effects of ETO (1 µM) in HCT-116 cells but not in HepG2. Synergism is postulated, but not shown. | [10] |
| Doxorubicin (Dox) | Cytotoxic effect of Dox-resistant B16 melanoma cell subline (B16/DOX) | The cytotoxicity of Dox (0.25–5 µM) is increased by 10–25 µM pretreatment with Resv. | [11] |
| Doxorubicin (Dox) | Cell viability of MCF-7 cells | Strong combinatory effect on cell viability by Resv (50–250 µM) and Dox (5 µM). | [12] |
| Doxorubicin (Dox) | Dox-resistant breast cancer  (MCF-7/adr) and MDA-MB-231 cells | Combination of Resv (50 µM) and Dox (1–10 µM) statistical significantly decreased cell viability. | [13] |
| Doxorubicin or Docetaxel (Doc) | Cell proliferation in MCF-7,  HeLa and HepG2 | Resv in combination with Dox and Doc increased their potencies showing IC_50_ ranging from 0.12 to 0.34 µM (Dox alone: 0.48 to 0.72 µM) and from 7.2 to 53 nM (Doc alone: 0.48 to 0.72 µM), respectively. | [14] |
| Fludarabine or Cladribine | Induction of apoptosis in chronic lymphocytic leukemia (CLL) cells. | Both Resv (40 µM) + fludarabine (1 μg/mL) and Resv + cladribine (1.4 μg/mL) caused synergistically higher apoptosis rates relative to the rates caused by the single drugs. | [15] |
| Gemcitabine | Modulation of cell proliferation, apoptotic effect of pancreas cells and effect in a mouse orthotopic model of human PaCa cells. | Combination of Resv (10 µM) and gemcitabine (100 µM) cause a significant apoptosis, but is not observed by the single compounds. In the mouse orthotopic model, the combination showed stronger response relative to the single compounds. | [16] |
| 5-Fluorouracil (5-FU) | Cell proliferation viability and apoptosis in HCT-116 cells | A significant inhibition of cell proliferation, migration, and  increased apoptosis was observed with 15 µM Resv in combination with 0.5 µM 5-FU. | [17] |
| 5-fluorouracil (5-FU) | Cell growth in colon cancer cell lines DLD-1, SW480 and COLO201 | Synergistic action of Resv (10 µM) and 5-FU (1 µM) in DLD-1 and SW480 and additive effect in COLO201 | [18] |
| Clofarabine | Viability of malignant mesothelioma MSTO-211H cells or normal mesothelial MeT-5A cells. | Synergistic response of combination of Resv (10–20 µM) and Clofarabine (40 nM) was observed in MSTO-211H. Marginal effects were observed on normal cells. | [19,20] |
| Decitabine (Dec) | HIV-1 infectivity | A more than additive effect of Resv (20 µM) and Dec (70 nM) in combination reduction of HIV-1 infectivity | [21] |
| Cyclophosphamide | 5 mM CPA and 50 µM RES in MCF-7 in induction of apoptosis | Combinatory effect on apoptosis | [22] |
| Temozolomide (TMZ) | TMZ-induced cell death (U87MG cells) | Combination of TMZ (100–400 μM) and Resv (10 µM) has a synergistic effect on cell death. | [23] |
| Temozolomide (TMZ) | TMZ resistance | Combining TMZ (100 µM) with Resv (100 µM) resulted in a reduced cell viability by TMZ, and an increase the apoptosis in TMZ-resistant T98G cells. | [24] |
| Temozolomide (TMZ) | Cell survival of SHG44 cells after 72 h. | The combination of TMZ (100 µM) and Resv (10 µM) causes a significant less cell survival. | [25] |
| Temozolomide (TMZ) | Modulation of autophagy, apoptosis, cell cycle dynamics, senescence induction and clonogenic capacity in human glioma cells (U87). | Resv (30–100 μM) potentiates the cytotoxic effects of TMZ (100–1000 μM) in human glioma cells, but Resv abrogates TMZ-induced cell cycle arrest. Therefore, Resv leads to mitotic catastrophe and the combination causes a reduction in clonogenic capacity, accompanied by a large induction of senescence. | [26] |
| Melphalan (MEL) | Enhance the antitumor effects of MEL in MCF-7 and MDA-MB-231 cells. | The reduced viability of the two cell lines by combinations of MEL (25–75 μM) and Resv (50–150 μM) for 24 h was higher than with the two compounds alone. Treatment with Resv first followed by MEL gave a stronger effect compared with opposite treatment regime. | [27] |
| 1,3-Bis(2-chloro-ethyl)-1-nitrosourea (Carmustine, BCNU) | Cell viability and apoptosis in 5-FU sensitive and resistant HCT-116 cells. | Combined application of BCNU (20 µM) and Resv (10–50 µM) reduces cell viability and increases apoptosis in 5-FU sensitive cells relative to individual treatments. In 5-FU resistant cells, addition of BCNU reduces the IC_50_ for Resv. Induction of apoptosis was synergistic by Resv and BCNU. | [28] |
| Arabino-Furano-sylcytosine (Ara-C) | Sequential growth inhibition in human HL-60 promyelocytic leukemia. | Additive effects of Digalloylresveratrol (DIG, 4–5 µM) and Ara-C (10–20 nM) were observed on the cell proliferation. HL-60 cells were treated with DIG for 24 h and thereafter with Ara-C for 72 h. | [29] |
| Proteasome inhibitor, MG132 | Sensitize leukemic cells (K562, U937, NB4, Daudi and Raji cells) to proteasome inhibitors | Resv (1–20 µM) strongly reduced cytotoxic activities of MG132 (5 µM) against leukemic cells. Resv reduced the MG132-induced apoptosis. | [30] |
| Diethylstilbestrol (DES) | Modulation of the DES-DNA adduct formation. | In MCF-10F cells, Resv (3–30 µM) reduces the level of DES-DNA adduct formation (by 10 µM DES). | [31] |
| Flutamide | Effect on androgen receptor (AR) and PSA levels in DHT induced LNCaP cells. | The combination of Resv (50 µM) with flutamide (100 nM) had a synergistic effect on down-regulation of androgen receptor. | [32] |
| Combination of RQC (Resv + quercetin + catechin) on Gefitinib | MDA-MB-231 cell viability. | IC_50_ for RQC and Gefitinib is 2.4 µM and 24 µM, respectively, and the same response may be found with the combination of 10 µM Gefitinib and 5 µM RQC or combination of 30 µM Gefitinib and 1.67 µM RQC. | [33] |
| Arsenic trioxide | Viability, apoptosis and colony formation of acute myeloid leukemia (AML) or chronic myeloid leukemia (CML) cells. | In all parameters, Resv (10–25 µM) potentiates the effects of arsenic trioxide (0.25–0.5 µM). | [34] |
| 4-*n*-butylresorcinol | Inhibition of tyrosinase activity and melanin synthesis in Mel-Ab cells. | At 1 µM neither 4-*n*-butylresorcinol nor Resv did inhibit melanin synthesis but only their combination. Synergistic effect is reported, but such data are not shown. | [35] |
| Simvastatin (SIM) | Cholesterol synthesis and HMGCR enzyme activity in human endometrial stromal cells | Resv (30 µM) potentiated the inhibitory effects of SIM (0.1 µM) on cholesterol biosynthesis and HMGCR enzyme activity (HMGCR = 3-hydroxy-3-methylglutaryl-coenzyme A reductase). | [36] |
| Simvastatin (SIM) | Isolated rat theca-interstitial cells | Resv (3–10 µM) potentiated inhibitory effects of simvastatin (1 µM) on androstenedione and androsterone production. | [37] |
| Metformin (Met) and hydroxyl-methyl-butyrate (HMB) | AMP-activated protein kinase-  Dependent in C2C12 skeletal myotubes and 3T3-L1 adipocytes | Resv + Met + HMB (0.2 µM + 0.1 mM + 5 µM) significantly increased fat oxidation, AMP-activated protein kinase, and Sirt1 activity in C2C12 cells compared with Met or Resv + HMB alone. Similar trend was observed in 3T3-L1 adipocytes. | [38] |
| Ionizing radiation (IR) | Toxicity of IR in prostate cell lines | Pretreatment with Resv (2.5–10 µM) inhibited clonogenic survival of PC3 and 22RV1 cells. | [39] |
| Ionizing radiation (IR) | Clonogenic assays using A549 and H460 | Resv (20 µM) enhances IR-induced cell killing in lung cancer cells. | [40] |
| Radiation therapy (XRT) | Radiosensitizing a radioresistant prostate cancer cell line, PC-3. | Combination of Resv (2–50 µM) with XRT synergistically enhanced the XRT-induced apoptosis and cell proliferation inhibition of PC-3 cells. | [41,42] |
| Radiation therapy (XRT) | Effects of XRT in combination with RSV on radio-resistant melanoma lines, SK-Mel-5 and HTB-65 | Resv (2–50 µM) enhances radiation sensitivity of melanoma cells by inhibiting proliferation and promoting apoptosis. | [43] |

**Table S2.** Effects of various drugs *in vivo* modulated by resveratrol (Resv).

| **Compound** | **Model** | **Effects** | **Reference** |
| --- | --- | --- | --- |
| Cisplatin (CIP) | Prevention of cisplatin-related ototoxicity | CIP-related (a single dose, 12 mg/kg CIP i.p.) ototoxicity was prevented by Resv use (10 mg/kg i.p. for 5 days). | [44] |
| Cisplatin (CIP) | Prevention of recurrent tumor cell growth (A2780 ovarian carcinoma Cells) in Female nu/nu mice. | Mice treated with Resv (160 mg/kg i.p. every day × 14 days) after treatment with CIP (6 mg/kg i.p. every 3 days × 3), did have reduced tumor growth compared with mice treated with vehicle control. Resv and CIP treatment was initiated on day 5 after tumor cell injection. | [45] |
| Cisplatin (CIP) | Anti-tumor effect after sub-cutaneous injection of B16F10 tumor cells into C57BL/6 mice | Tumor size was reduced and survival rate was extended by combination of CIP (4 mg/kg i.p. three times a week) and Resv (10 mg/kg p.o. daily for seven days) compared to the single substances. | [46] |
| Cisplatin (CIP) | Ehrlich ascites carcinoma solid tumor model in Female Swiss albino mice. | The anti-tumor effects for the combination of CIP (5 mg/kg, i.p. daily for 20 days) and Resv (20 mg/kg, i.p. for 20 days) were significantly higher than for CIP and Resv alone. | [47] |
| Cisplatin (CIP) in combination with piceatannol (PIC) | Tumor growth in a ovarian cancer mouse model (male athymic nude mice). | A significant interaction was observed for PIC (20 mg/kg/day, 5 days/week, i.p.) and CIP (1.8 mg/kg/week, i.p.) in the reduction of tumor volume and reduced tumor size. | [48] |
| Doxorubicine (DOX) | Protection of the cardio-toxic effect of DOX (male wild-type Balb/c mice). | Resv (in the diet approx. intake of 15 mg/kg b.w. each day) counteracts the DOX-induced (8 mg/kg i.p. every 3rd week, 3×) serum lactate dehydrogenase, and DOX-induced mitochondrial cytochrome c release and cardiomyocyte apoptosis. | [49] |
| Doxorubicin (DOX) | Cardiomyocyte apoptosis in male BALB/c nude mice | Resv (10 mg/kg/day p.o.) diminished DOX-induced (2 mg/kg b.w. six times for 2 weeks) losses of the body-and heart weight. Resv reduced the DOX-induced apoptosis. | [50] |
| Doxorubicin (DOX) | Nude mouse xenograft model (female BALB/c athymic nude mice) injected MCF-7/adr cells. | Combination of Resv (20 mg/kg p.o. for four weeks) and Dox (4 mg/kg once a week for four weeks) inhibited tumor volume by 60% relative to control with only marginal effects of DOX. | [13] |
| Temozolomide (TMZ) | *In vivo* mouse xenograft (female nude mice (BALB/c nu/nu) injected U87 MG cells. | Co-administration of Resv (12.5 mg/kg s.c. daily for 12 days) and TMZ (10 mg/kg s.c. daily for 12 days) reduced tumor volumes significant more than Resv and TMZ alone. | [23] |
| Temozolomide (TMZ) | Glioblastoma orthotopic xenografts in Female BALB/cA nude mice (SHG44 cells). | The combination of TMZ (68 mg/kg p.o. for 3 days) and Resv (40 mg/kg p.o. daily dose, 20 days) caused a significant reduced tumor size and reduced Ki-67 staining. | [25] |
| Diclofenac or Benfotiamine | Anti-nociceptive interactions (female ICR mice) | Diclofenac (1.2–56.2 mg/kg, p.o.) and Resv (10–316.2 mg/kg, i.p.) or Benfotiamine (100–707.9 mg/kg, p.o.) and Resv combinations interact synergistically to reverse acetic acid-induced nociception. | [51] |
| Propofol | Hepatocyte apoptosis in rats with hepatic ischemia-reperfusion injury. | Propofol (10 or 20 mg·kg^−1^·h^−1^) and Resv (10 or 20 mg/kg) alone or in combination all significantly alleviated the hepatic pathologies, lowered the apoptosis index. | [52] |
| Atorvastatin | Anti-atherogenic effect of mild statin treatment in female APOE*3-Leiden.CETP mice. | Resv (0.01% *w*/*w*; 11 mg/kg/day) does not add to the anti-atherogenic effect of atorvastatin (0.0018% *w*/*w*; 2 mg/kg/day, increased to 0.0027% *w*/*w*; 3 mg/kg/day after four weeks) when treated in combination. | [53] |
| Metformin and hydroxymethylbutyrate (HMB) | Insulin sensitivity db/db mice | By combining low metformin exposures (0.25 or 0.75 g/kg diet) with Resv (12.5 mg/kg diet) + HMB (2 g/kg diet) significantly reduce the insulin sensitivity in mice. | [38] |
| Phenelzine | Mice (C57Bl6/J wild-type) were fed a very-high-fat diet (VHFD) | The altered glucose tolerance and the increased fat body composition of VHFD-fed mice were not reversed by Resv (0.003% in the drinking water) and/or phenelzine (0.02% in the drinking water). | [54] |
| Hydralazine | Hypertension-induced cardiovascular dysfunction in spontaneously hypertensive rats (Wistar–Kyoto rats) | Combination of Resv (2.5 mg/kg per day) with hydralazine (25 mg/kg per day) for eight weeks, was more effective than Resv or hydralazine alone in improving the overall cardiovascular parameters (blood pressure, cardiac structure and function). | [55] |

References

1. Mao, Q.Q.; Bai, Y.; Lin, Y.W.; Zheng, X.Y.; Qin, J.; Yang, K.; Xie, L.P. Resveratrol confers resistance against taxol via induction of cell cycle arrest in human cancer cell lines. *Mol. Nutr. Food Res.* **2010**, *54*, 1574–1584.
2. Zhan, Y.; Chen, Y.; Liu, R.; Zhang, H.; Zhang, Y. Potentiation of paclitaxel activity by curcumin in human breast cancer cell by modulating apoptosis and inhibiting EGFR signaling. *Arch. Pharm. Res.* **2014**, 37, 1086–1095.
3. Olgun, Y.; Altun, Z.; Aktas, S.; Ercetin, P.; Kirkim, G.; Kiray, M.; Bagriyanik, A.; Ozogul, C.; Serbetcioglu, B.; Guneri, E.A. Molecular mechanisms of protective effect of resveratrol against cisplatinium induced ototoxicity. *J. Int. Adv. Otol.* **2013**, *9*, 145–152.
4. Nessa, M.U.; Beale, P.; Chan, C.; Yu, J.Q.; Huq, F. Combinations of resveratrol, cisplatin and oxaliplatin applied to human ovarian cancer cells. *Anticancer Res.* **2012**, *32*, 53–59.
5. Kaminski, B.M.; Weigert, A.; Scherzberg, M.C.; Ley, S.; Gilbert, B.; Brecht, K.; Brune, B.; Steinhilber, D.; Stein, J.; Ulrich-Ruckert, S. Resveratrol-induced potentiation of the antitumor effects of oxaliplatin is accompanied by an altered cytokine profile of human monocyte-derived macrophages. *Apoptosis* **2014**,
   *19*, 1136–1147.
6. Bjorklund, M.; Roos, J.; Gogvadze, V.; Shoshan, M. Resveratrol induces SIRT1- and energy-stress-independent inhibition of tumor cell regrowth after low-dose platinum treatment. *Cancer Chemother. Pharmacol.* **2011**, 68, 1459–1467.
7. Hu, F.W.; Tsai, L.L.; Yu, C.H.; Chen, P.N.; Chou, M.Y.; Yu, C.C. Impairment of tumor-initiating stem-like property and reversal of epithelial-mesenchymal transdifferentiation in head and neck cancer by resveratrol treatment. *Mol. Nutr. Food Res.* **2012**, *56*, 1247–1258.
8. Huq, F.; Yu, J.Q.; Beale, P.; Chan, C.; Arzuman, L.; Nessa, M.U.; Mazumder, M.E.H. Combinations of platinums and selected phytochemicals as a means of overcoming resistance in ovarian cancer. *Anticancer Res.* **2014**, *34*, 541–545.
9. Heiduschka, G.; Lill, C.; Seemann, R.; Brunner, M.; Schmid, R.; Houben, R.; Bigenzahn, J.; Thurnher, D. The effect of resveratrol in combination with irradiation and chemotherapy: Study using Merkel cell carcinoma cell lines. *Strahlenther. Onkol.* **2014**, *190*, 75–80.
10. Amiri, F.; Zarnani, A.H.; Zand, H.; Koohdani, F.; Jeddi-Tehrani, M.; Vafa, M. Synergistic anti-proliferative effect of resveratrol and etoposide on human hepatocellular and colon cancer cell lines. *Eur. J. Pharmacol.* **2013**, *718*, 34–40.
11. Gatouillat, G.; Balasse, E.; Joseph-Pietras, D.; Morjani, H.; Madoulet, C. Resveratrol induces cell-cycle disruption and apoptosis in chemoresistant B16 melanoma. *J. Cell. Biochem.* **2010**, *110*, 893–902.
12. Diaz-Chavez, J.; Fonseca-Sanchez, M.A.; Arechaga-Ocampo, E.; Flores-Perez, A.; Palacios-Rodriguez, Y.; Dominguez-Gomez, G.; Marchat, L.A.; Fuentes-Mera, L.; Mendoza-Hernandez, G.; Gariglio, P.; *et al.* Proteomic profiling reveals that resveratrol inhibits HSP27 expression and sensitizes breast cancer cells to doxorubicin therapy. *PLoS ONE* **2013**, *8*, e64378.
13. Kim, T.H.; Shin, Y.J.; Won, A.J.; Lee, B.M.; Choi, W.S.; Jung, J.H.; Chung, H.Y.; Kim, H.S. Resveratrol enhances chemosensitivity of doxorubicin in multidrug-resistant human breast cancer cells via increased cellular influx of doxorubicin. *Biochim. Biophys. Acta* **2014**, *1840*, 615–625.
14. Al-Abd, A.M.; Mahmoud, A.M.; El-Sherbiny, G.A.; El-Moselhy, M.A.; Nofal, S.M.; El-Latif, H.A.; El-Eraky, W.I.; El-Shemy, H.A. Resveratrol enhances the cytotoxic profile of docetaxel and doxorubicin in solid tumour cell lines *in vitro*. *Cell Prolif.* **2011**, *44*, 591–601.
15. Podhorecka, M.; Halicka, D.; Klimek, P.; Kowal, M.; Chocholska, S.; Dmoszynska, A. Resveratrol increases rate of apoptosis caused by purine analogues in malignant lymphocytes of chronic lymphocytic leukemia. *Ann. Hematol.* **2011**, *90*, 173–183.
16. Harikumar, K.B.; Kunnumakkara, A.B.; Sethi, G.; Diagaradjane, P.; Anand, P.; Pandey, M.K.; Gelovani, J.; Krishnan, S.; Guha, S.; Aggarwal, B.B. Resveratrol, a multitargeted agent, can enhance antitumor activity of gemcitabine *in vitro* and in orthotopic mouse model of human pancreatic cancer. *Int. J. Cancer* **2010**,
    *127*, 257–268.
17. Mohapatra, P.; Preet, R.; Choudhuri, M.; Choudhuri, T.; Kundu, C.N. 5-fluorouracil increases the chemopreventive potentials of resveratrol through DNA damage and MAPK signaling pathway in human colorectal cancer cells. *Oncol. Res.* **2011**, *19*, 311–321.
18. Kumazaki, M.; Noguchi, S.; Yasui, Y.; Iwasaki, J.; Shinohara, H.; Yamada, N.; Akao, Y. Anti-cancer effects of naturally occurring compounds through modulation of signal transduction and miRNA expression in human colon cancer cells. *J. Nutr. Biochem.* **2013**, *24*, 1849–1858.
19. Lee, Y.J.; Lee, Y.J.; Im, J.H.; Won, S.Y.; Kim, Y.B.; Cho, M.K.; Nam, H.S.; Choi, Y.J.; Lee, S.H. Synergistic anti-cancer effects of resveratrol and chemotherapeutic agent clofarabine against human malignant mesothelioma MSTO-211H cells. *Food Chem. Toxicol.* **2013**, *52*, 61–68.
20. Lee, Y.J.; Im, J.H.; Lee, D.M.; Park, J.S.; Won, S.Y.; Cho, M.K.; Nam, H.S.; Lee, Y.J.; Lee, S.H. Synergistic inhibition of mesothelioma cell growth by the combination of clofarabine and resveratrol involves Nrf2 downregulation. *BMB Rep.* **2012**, *45*, 647–652.
21. Clouser, C.L.; Chauhan, J.; Bess, M.A.; Oploo, J.L.; Zhou, D.; Dimick-Gray, S.; Mansky, L.M.; Patterson, S.E. Anti-HIV-1 activity of resveratrol derivatives and synergistic inhibition of HIV-1 by the combination of resveratrol and decitabine. *Bioorg. Med. Chem. Lett.* **2012**, *22*, 6642–6646.
22. Singh, N.; Nigam, M.; Ranjan, V.; Zaidi, D.; Garg, V.K.; Sharma, S.; Chaturvedi, R.; Shankar, R.; Kumar, S.; Sharma, R.; *et al.* Resveratrol as an adjunct therapy in cyclophosphamide-treated MCF-7 cells and breast tumor explants. *Cancer Sci.* **2011**, *102*, 1059–1067.
23. Lin, C.J.; Lee, C.C.; Shih, Y.L.; Lin, T.Y.; Wang, S.H.; Lin, Y.F.; Shih, C.M. Resveratrol enhances the therapeutic effect of temozolomide against malignant glioma *in vitro* and *in vivo* by inhibiting autophagy. *Free Radic. Biol. Med.* **2012**, *52*, 377–391.
24. Huang, H.; Lin, H.; Zhang, X.; Li, J. Resveratrol reverses temozolomide resistance by downregulation of MGMT in T98G glioblastoma cells by the NF-kappaB-dependent pathway. *Oncol. Rep.* **2012**, *27*, 2050–2056.
25. Yuan, Y.; Xue, X.; Guo, R.B.; Sun, X.L.; Hu, G. Resveratrol enhances the antitumor effects of temozolomide in glioblastoma via ROS-dependent AMPK-TSC-mTOR signaling pathway. *CNS Neurosci. Ther.* **2012**,
    *18*, 536–546.
26. Filippi-Chiela, E.C.; Thome, M.P.; Bueno e Silva, M.M.; Pelegrini, A.L.; Ledur, P.F.; Garicochea, B.; Zamin, L.L.; Lenz, G. Resveratrol abrogates the temozolomide-induced G2 arrest leading to mitotic catastrophe and reinforces the temozolomide-induced senescence in glioma cells. *BMC Cancer* **2013**, *13*, 147.
27. Casanova, F.; Quarti, J.; da Costa, D.C.; Ramos, C.A.; da Silva, J.L.; Fialho, E. Resveratrol chemosensitizes breast cancer cells to melphalan by cell cycle arrest. *J. Cell. Biochem.* **2012**, *113*, 2586–2596.
28. Das, D.; Preet, R.; Mohapatra, P.; Satapathy, S.R.; Kundu, C.N. 1,3-Bis(2-chloroethyl)-1-nitrosourea enhances the inhibitory effect of resveratrol on 5-fluorouracil sensitive/resistant colon cancer cells. *World J. Gastroenterol.* **2013**, *19*, 7374–7388.
29. Saiko, P.; Graser, G.; Madlener, S.; Schwarz, S.; Krupitza, G.; Jaeger, W.; Somepalli, V.; Golakoti, T.; Fritzer-Szekeres, M.; Szekeres, T. Combination effects of digalloylresveratrol with arabinofuranosylcytosine and difluorodeoxycytidine in human leukemia and pancreatic cancer cells. *Nucleosides Nucleotides Nucl. Acids* **2011**, *30*, 1190–1196.
30. Niu, X.F.; Liu, B.Q.; Du, Z.X.; Gao, Y.Y.; Li, C.; Li, N.; Guan, Y.; Wang, H.Q. Resveratrol protects leukemic cells against cytotoxicity induced by proteasome inhibitors via induction of FOXO1 and p27Kip1. *BMC Cancer* **2011**, *11*, 99.
31. Hinrichs, B.; Zahid, M.; Saeed, M.; Ali, M.F.; Cavalieri, E.L.; Rogan, E.G. Formation of diethylstilbestrol-DNA adducts in human breast epithelial cells and inhibition by resveratrol. *J. Steroid Biochem. Mol. Biol.* **2011**, *127*, 276–281.
32. Kai, L.; Levenson, A.S. Combination of resveratrol and antiandrogen flutamide has synergistic effect on androgen receptor inhibition in prostate cancer cells. *Anticancer Res.* **2011**, *31*, 3323–3330.
33. Castillo-Pichardo, L.; Dharmawardhane, S.F. Grape polyphenols inhibit Akt/mammalian target of rapamycin signaling and potentiate the effects of gefitinib in breast cancer. *Nutr. Cancer* **2012**, *64*, 1058–1069.
34. Wu, E.J.; Goussetis, D.J.; Beauchamp, E.; Kosciuczuk, E.M.; Altman, J.K.; Eklund, E.A.; Platanias, L.C. Resveratrol enhances the suppressive effects of arsenic trioxide on primitive leukemic progenitors. *Cancer Biol. Ther.* **2014**, *15*, 473–478.
35. Kim, S.Y.; Park, K.C.; Kwon, S.B.; Kim, D.S. Hypopigmentary effects of 4-*n*-butylresorcinol and resveratrol in combination. *Pharmazie* **2012**, *67*, 542–546.
36. Villanueva, J.A.; Sokalska, A.; Cress, A.B.; Ortega, I.; Bruner-Tran, K.L.; Osteen, K.G.; Duleba, A.J. Resveratrol potentiates effect of simvastatin on inhibition of mevalonate pathway in human endometrial stromal cells. *J. Clin. Endocrinol. MeTable* **2013**, *98*, E455–E462.
37. Ortega, I.; Villanueva, J.A.; Wong, D.H.; Cress, A.B.; Sokalska, A.; Stanley, S.D.; Duleba, A.J. Resveratrol potentiates effects of simvastatin on inhibition of rat ovarian theca-interstitial cells steroidogenesis. *J. Ovarian Res.* **2014**, *7*, 21.
38. Bruckbauer, A.; Zemel, M.B. Synergistic effects of metformin, resveratrol, and hydroxymethylbutyrate on insulin sensitivity. *Diabetes Metab. Syndr. Obes.* **2013**, *6*, 93–102.
39. Rashid, A.; Liu, C.; Sanli, T.; Tsiani, E.; Singh, G.; Bristow, R.G.; Dayes, I.; Lukka, H.; Wright, J.; Tsakiridis, T. Resveratrol enhances prostate cancer cell response to ionizing radiation. Modulation of the AMPK, Akt and mTOR pathways. *Radiat. Oncol.* **2011**, *6*, 144.
40. Luo, H.; Wang, L.; Schulte, B.A.; Yang, A.; Tang, S.; Wang, G.Y. Resveratrol enhances ionizing radiation-induced premature senescence in lung cancer cells. *Int. J. Oncol.* **2013**, *43*, 1999–2006.
41. Fang, Y.; Demarco, V.G.; Nicholl, M.B. Resveratrol enhances radiation sensitivity in prostate cancer by inhibiting cell proliferation and promoting cell senescence and apoptosis. *Cancer Sci.* **2012**, *103*, 1090–1098.
42. Fang, Y.J.; Herrick, E.J.; Nicholl, M.B. A possible role for perforin and granzyme B in resveratrol-enhanced radiosensitivity of prostate cancer. *J. Androl.* **2012**, *33*, 752–760.
43. Fang, Y.; Bradley, M.J.; Cook, K.M.; Herrick, E.J.; Nicholl, M.B. A potential role for resveratrol as a radiation sensitizer for melanoma treatment. *J. Surg. Res.* **2013**, *183*, 645–653.
44. Erdem, T.; Bayindir, T.; Filiz, A.; Iraz, M.; Selimoglu, E. The effect of resveratrol on the prevention of cisplatin ototoxicity. *Eur. Arch. Otorhinolaryngol.* **2012**, *269*, 2185–2188.
45. Tan, L.; Wang, W.; He, G.; Kuick, R.D.; Gossner, G.; Kueck, A.S.; Wahl, H.; Opipari, A.W.; Liu, J.R. Resveratrol inhibits ovarian tumor growth in an *in vivo* mouse model. *Cancer* **2016**, *122*, 722–729.
46. Cheng, Y.J.; Chang, M.Y.; Chang, W.W.; Wang, W.K.; Liu, C.F.; Lin, S.T.; Lee, C.H. Resveratrol enhances chemosensitivity in mouse melanoma model through connexin 43 upregulation. *Environ. Toxicol.* **2014**,
    *30*, 877–886.
47. El-Mowafy, A.M.; El-Mesery, M.E.; Salem, H.A.; Al-Gayyar, M.M.; Darweish, M.M. Prominent chemopreventive and chemoenhancing effects for resveratrol: Unraveling molecular targets and the role of C-reactive protein. *Chemotherapy* **2010**, *56*, 60–65.
48. Farrand, L.; Byun, S.; Kim, J.Y.; Im-Aram, A.; Lee, J.; Lim, S.; Lee, K.W.; Suh, J.Y.; Lee, H.J.; Tsang, B.K. Piceatannol enhances cisplatin sensitivity in ovarian cancer via modulation of p53, X-linked inhibitor of apoptosis protein (XIAP), and mitochondrial fission. *J. Biol. Chem.* **2013**, *288*, 23740–23750.
49. Zhang, C.; Feng, Y.S.; Qu, S.L.; Wei, X.; Zhu, H.L.; Luo, Q.; Liu, M.D.; Chen, G.W.; Xiao, X.Z. Resveratrol attenuates doxorubicin-induced cardiomyocyte apoptosis in mice through SIRT1-mediated deacetylation of p53. *Cardiovasc. Res.* **2011**, *90*, 538–545.
50. Gu, J.; Song, Z.P.; Gui, D.M.; Hu, W.; Chen, Y.G.; Zhang, D.D. Resveratrol attenuates doxorubicin-Induced cardiomyocyte apoptosis in lymphoma nude mice by heme oxygenase-1 induction. *Cardiovasc. Toxicol.* **2012**, *12*, 341–349.
51. Montiel-Ruiz, R.M.; Granados-Soto, V.; Garcia-Jimenez, S.; Reyes-Garcia, G.; Flores-Murrieta, F.J.; Deciga-Campos, M. Synergistic interaction of diclofenac, benfotiamine, and resveratrol in experimental acute pain. *Drug Dev. Res.* **2011**, *72*, 397–404.
52. Shen, X.; Zhao, G.; Wang, R.; Liu, T.; Liu, L. Protective effect of propofol and resveratrol pretreatment against hepatocyte apoptosis in rats with hepatic ischemia-reperfusion injury. *Nan Fang Yi Ke Da Xue Xue Bao* **2013**, *33*, 80–85.
53. Berbee, J.F.; Wong, M.C.; Wang, Y.; van der Hoorn, J.W.; Khedoe, P.P.; van Klinken, J.B.; Mol, I.M.; Hiemstra, P.S.; Tsikas, D.; Romijn, J.A*.*; *et al.* Resveratrol protects against atherosclerosis, but does not add to the antiatherogenic effect of atorvastatin, in APOE*3-Leiden.CETP mice. *J. Nutr. Biochem.* **2013**, *24*, 1423–1430.
54. Carpene, C.; Gomez-Zorita, S.; Gupta, R.; Gres, S.; Rancoule, C.; Cadoudal, T.; Mercader, J.; Gomez, A.; Bertrand, C.; Iffiu-Soltesz, Z. Combination of low dose of the anti-adipogenic agents resveratrol and phenelzine in drinking water is not sufficient to prevent obesity in very-high-fat diet-fed mice. *Eur. J. Nutr.* **2014**, *53*, 1625–1635.
55. Thandapilly, S.J.; Louis, X.L.; Behbahani, J.; Movahed, A.; Yu, L.; Fandrich, R.; Zhang, S.; Kardami, E.; Anderson, H.D.; Netticadan, T. Reduced hemodynamic load aids low-dose resveratrol in reversing cardiovascular defects in hypertensive rats. *Hypertens. Res.* **2013**, *36*, 866–872.
