# Supplementary material for: Challenges in Analyzing the Biological Effects of Resveratrol
Source: Nutrients. 2016 Jun 9;8(6):353. doi: 10.3390/nu8060353 (PMC4924194; doi:10.3390/nu8060353)
Supplement: Supplementary file 1 [file nutrients-08-00353-s001.zip › nutrients-126957 Suppl Table 05-final.docx]

Supplementary Materials: Challenges in Analyzing the Biological Effects of Resveratrol

Cihan Suleyman Erdogan and Ole Vang

**Table S5.** Effects of various mixtures containing resveratrol (Resv).

| **Compounds** | **Activity** | **Effect** | **Reference** |
| --- | --- | --- | --- |
| *In vitro* studies | | | |
| Combination of a mixture of Resv, epicatechin, cyanidin and quercetin (QUE) in comparison with the single compounds and grape powder. | Cell viability, inhibition of taurodeoxycholic acid-induced ROS and apoptosis in Huh7 cells. | The preventive effect of 300 µg/mL grape powder was observed by the mixture of Resv (25 µM), epicatechin (100 µM), anthocyanin (50 µM), and QUE (100 µM). The single components did not show any effect. | [1] |
| Mixture of ferulic acid, phloretin, curcumin (CUR), and Resv | Migration of primary human gingival fibroblasts and human periodontal ligament fibroblast. | Combination of two or three substances (all as 10 µM) was generally more effective than single compounds. | [2] |
| Mixtures: RFT: Resv, ferulic acid and curcumin (CUR); PFR: phloretin, ferulic acid and Resv; PFT: phloretin, ferulic acid and CUR, all 1:1:1 by weight ratio composition | Cell viability and DNA synthesis in human gingival fibroblasts | Treatment with H_2_O_2_, EtOH and nicotine caused decreased cell viability and reduced DNA synthesis. Reduction of these biomarkers were counteracted by all three mixtures. | [3] |
| Mixtures: RFT: Resv, ferulic acid and curcumin (CUR); PFR: phloretin, ferulic acid and Resv; PFT: phloretin, ferulic acid and CUR, all 1:1:1 by weight ratio composition | Cu and Zn-induced cytotoxicity of human gingival fibroblasts and human periodontal ligament cells. | The mixtures increased recovery of cells exposed to Cu and Zn. | [4] |
| Animal studies | | | |
| Mixtures of apigenin (API), baicalein (BAI), curcumin (CUR), epigallocatechin 3-gallate (EGCG), genistein (GEN), Quercetin (QUE), and Resv | Cancer treatment and prevention abilities in transgenic adenocarcinoma of the mouse prostate (TRAMP) mice. | Mixtures of CUR (10 mg/kg diet) + EGCG (100 mg/kg diet) + Resv (10 mg/kg diet), mixtures of API (15 mg/kg diet) + BAI (150 mg/kg diet) + GEN (250 mg/kg diet) + QUE (1 g/kg diet), as well all seven compounds together were able to reduce or delay prostate cancer *in vivo*. | [5] |
| Black Tea Polyphenol (BTP) | Suppression of the two-stage mouse skin carcinogenesis induced by DMBA and TPA (male, Balb/c mice). | Resv- (0.01 µmol topical applicated) or BTP- (0.2% in drinking water, three times per week) alone treatment decreased tumor incidence, while their combination at low doses synergistically decreased tumor incidence even more. | [6] |
| Grape seed extract (GSE), concord grape juice extract in combination with Resv | Amyloid-β (Aβ) mediated neuropathology and cognitive impairments in a mouse model of AD (J20 AD mice). | Combination treatment resulted in better protection against cognitive impairments compared to individual treatments. Resv (400 mg/kg mixed with food), GSE (200 mg/kg mixed with food); the juice extract: 183 mg total polyphenol/kg food. | [7] |
| Nutrient supplementation, containing (mg/kg/day): R-α-lipoic acid (50), acetyl-l-carnitine (100), biotin (0.1), nicotinamide (15), riboflavin (6), pyridoxine (6), creatine (50), CoQ10 (5), Resv (5) and taurine (100). | Effect on running distances, exercise-induced oxidative damage and mitochondrial dysfunction in male Sprague Dawley rats. | Nutrient supplementation caused significantly longer run distance, reduced exhaustive exercise-induced oxidative damage, and reduced mitochondrial dysfunction. | [8] |
| Human studies | | | |
| Resv, pterostilbene, morin hydrate, quercetin (QUE), δ-tocotrienol, riboflavin.  (NS-7 = 25 mg of each, Resv, pterostilbene, QUE, δ-tocotrienol, nicotinic acid, and morin hydrate or NS-6 = morin hydrate replaced with QUE, 50 mg QUE/capsule) | Elderly human subjects with normal or elevated serum cholesterol. Measure the effect on fatty acid and inflammation biomarkers. | Two capsules/day of NS-7 or NS-6 for four weeks reduced serum nitric oxide (NO) as well as the inflammation markers. | [9] |
| Nutritional Supplement-5 (NS-5: Resv, Pterostilbene, Quercetin (QUE), δ-tocotrienol, nicotinic acid). NS-5 contains QUE (50 mg), δ-tocotrienol, Resv, pterostilbene, nicotinic acid (25 mg of each). | A double blind, randomized, placebo-controlled cross-over trial with free-living and hyperchole-sterolemic elderly subjects exposed to the NS-5. | Following the exposure to NS-5 in six weeks, the serum levels of NO, CRP, γ-GT activity and blood pressure were decreased in free-living healthy seniors. No effect was observed on the total, HDL-, LDL-cholesterol or triglycerides.  In a hypercholesterolemic sub group, serum levels of NO, CRP, γ-GT, total cholesterol, LDL-cholesterol, triglycerides were reduced by NS-5. | [10] |
| Epigallocatechin-gallate (EG), Resv and soy isoflavones (SI) *N* = 18 | Double-blind randomized placebo-controlled cross-over trial, 3 days exposure. | Increased energy expenditure by EG (282 mg/day). Fasting plasma free fatty acid was increased by EG + SI (80 mg/day) + Resv (200 mg/day). | [11] |
| A complex of Resv (100 mg), each of green, black, and white tea extracts (800 mg), pomegranate extract (250 mg), quercetin (650 mg), acetyl-l-carnitine (500 mg), lipoic acid (600 mg), curcumin (900 mg), sesamin (1 g), cinnamon bark extract (1.7 g), and fish oil (1.0 g) | 6-month randomized, single-blind controlled trial, including 54 non-obese subjects. | These supplements did not either alter arterial stiffness or endothelial function, body fat measured by DEXA, blood pressure, plasma lipids, glucose, insulin, IGF-1, nor markers of inflammation and oxidative stress. | [12] |

References

1. Yu, J.; Xu, Y.M.; Khaoustov, V.; Yoffe, B. Identification of components of grape powder with anti-apoptotic effects. *Toxicol. Ind. Health* **2011**, *27*, 19–28.
2. San Miguel, S.M.; Opperman, L.A.; Allen, E.P.; Zielinski, J.; Svoboda, K.K. Antioxidants counteract nicotine and promote migration via RacGTP in oral fibroblast cells. *J. Periodontol.* **2010**, *81*, 1675–1690.
3. San Miguel, S.M.; Opperman, L.A.; Allen, E.P.; Zielinski, J.; Svoboda, K.K. Bioactive polyphenol antioxidants protect oral fibroblasts from ROS-inducing agents. *Arch. Oral. Biol.* **2012**, *57*, 1657–1667.
4. San Miguel, S.M.; Opperman, L.A.; Allen, E.P.; Zielinski, J.E.; Svoboda, K.K. Antioxidant combinations protect oral fibroblasts against metal-induced toxicity. *Arch. Oral. Biol.* **2013**, *58*, 299–310.
5. Slusarz, A.; Shenouda, N.S.; Sakla, M.S.; Drenkhahn, S.K.; Narula, A.S.; MacDonald, R.S.; Besch-Williford, C.L.; Lubahn, D.B. Common botanical compounds inhibit the hedgehog signaling pathway in prostate cancer. *Cancer Res.* **2010**, *70*, 3382–3390.
6. George, J.; Singh, M.; Srivastava, A.K.; Bhui, K.; Roy, P.; Chaturvedi, P.K.; Shukla, Y. Resveratrol and black tea polyphenol combination synergistically suppress mouse skin tumors growth by inhibition of activated MAPKs and p53. *PLoS ONE* **2011**, *6*, e23395.
7. Wang, J.; Bi, W.N.; Cheng, A.; Freire, D.; Vempati, P.; Zhao, W.; Gong, B.; Janle, E.M.; Chen, T.Y.; Ferruzzi, M.G.; *et al.* Targeting multiple pathogenic mechanisms with polyphenols for the treatment of Alzheimer’s disease-experimental approach and therapeutic implications. *Front. Aging Neurosci.* **2014**, *6*, 42.
8. Sun, M.; Qian, F.; Shen, W.; Tian, C.; Hao, J.; Sun, L.; Liu, J. Mitochondrial nutrients stimulate performance and mitochondrial biogenesis in exhaustively exercised rats. *Scand. J. Med. Sci. Sports* **2012**, *22*, 764–775.
9. Qureshi, A.A.; Khan, D.A.; Mahjabeen, W.; Papasian, C.J.; Qureshi, N. Suppression of Nitric Oxide production and cardiovascular risk factors in healthy seniors and hypercholesterolemic subjects by a combination of polyphenols and vitamins. *J. Clin. Exp. Cardiol.* **2012**, *5*, 8.
10. Qureshi, A.A.; Khan, D.A.; Mahjabeen, W.; Papasian, C.J.; Qureshi, N. Nutritional Supplement-5 with a combination of proteasome inhibitors (resveratrol, quercetin, *g*-tocotrienol) modulate age-associated biomarkers and cardiovascular lipid parameters in human subjects. *J. Clin. Exp. Cardiol.* **2013**, doi:10.4172/2155-9880.1000238.
11. Most, J.; Goossens, G.H.; Jocken, J.W.; Blaak, E.E. Short-term supplementation with a specific combination of dietary polyphenols increases energy expenditure and alters substrate metabolism in overweight subjects. *Int. J. Obes.* **2014**, *38*, 698–706.
12. Soare, A.; Weiss, E.P.; Holloszy, J.O.; Fontana, L. Multiple dietary supplements do not affect metabolic and cardio-vascular health. *Aging* **2014**, *6*, 149–157.
