# Supplementary material for: Challenges in Analyzing the Biological Effects of Resveratrol
Source: Nutrients. 2016 Jun 9;8(6):353. doi: 10.3390/nu8060353 (PMC4924194; doi:10.3390/nu8060353)
Supplement: Supplementary file 1 [file nutrients-08-00353-s001.zip › nutrients-126957 Suppl Table 03-04 -final.docx]

Supplementary Materials: Challenges in Analyzing the Biological Effects of Resveratrol

Cihan Suleyman Erdogan and Ole Vang

**Table S3.** Effects of various naturally occurring compounds *in vitro* modulated by resveratrol (Resv).

| **Compounds** | **Activity** | **Effect** | **Reference** |
| --- | --- | --- | --- |
| Pterostilbene (PTE) and quercetin (QUE) | Antioxidant activities in human erythrocytes *in vitro* measuring as lipid oxidation. | Combinatory index (CI) showed synergistic effect of combination of Resv and QUE (total conc. below 5 µM) and Resv and PTE (total conc. below 60 µM). | [1] |
| Polydatin (POL) | Effect on TNFα, IL-6 and IL-8 mRNA expression in heat stressed HaCat cells. | Combinations of POL (44 µM) and Resv (44 µM) reduced the expression of the cytokines more than the single compounds, but the increased total amounts of the stilbenes in the combinations were not considered. | [2] |
| Oxyresveratrol or curcumin (CUR) | Antioxidant assay (TMPD oxidation). | Combination of Resv (5 µM) and CUR (5 µM) showed much stronger response compared with combination of Resv (5 µM) and oxyresveratrol (5 µM) or the single compounds in double amounts. | [3,4] |
| Curcumin (CUR) or chrysin | Cell proliferation assay in Caco-2 cells. | Additive effect of Resv (20 µM) and chrysin (160 µM), as well as Resv (20 µM) and CUR (20 µM) on reduced cell proliferation. | [5] |
| Curcumin (CUR) | Cell viability and apoptosis induction in Hepa1-6 cells | Resv (10–160 µM) and CUR (2.5–40 µM) inhibited the cell viability synergistically. Combination of CUR and Resv was also found more effective in inducing apoptosis than either agent alone. | [6] |
| Curcumin (CUR) | Cigarette smoke condensate mediated transformed breast epithelial cell, MCF-10A-Tr. | IC_50_ was reduced 10× the combination of Resv (3 µM) and Cur (3 µM) in relative to 30 µM of the single compounds alone. Resv + CUR combination caused increased apoptosis. | [7] |
| Sulforaphane | Cell proliferation, migration and death in human U251 glioma cells. | Stronger effect of their combination (25 µM + 25 µM) than the single compounds (at 25 µM and 25 µM) | [8] |
| Diallyl disulfide (DADS) and curcumin (CUR) | Inhibition of cell survival of malignant rhabdoid cell (SJ-RH4, RD/18) and osteosarcoma (Saos-2). | No additional effect was obtained by the combination compared with the effects from the single compounds. All compounds were tested at 6 to 50 µM. | [9] |
| Quercetin, ethyl gallate, and (+)-catechin | Anti-proliferative response in vascular smooth muscle cell (VSMC). | All combinations including Resv (combinations of two-four compound) showed a synergistic response on serum induced cell proliferation. Equimolar combinations (final mixture concentration 10–100 µM) were used. | [10] |
| Genistein (GEN) | Cell viability and apoptosis induction in HeLa cells. | Resv (5 µM) and GEN (5 µM), when combined, can reduce cell viability and induce apoptosis slightly stronger than exposure to the single compounds (25 µM). | [11] |
| Chlorogenic acid (CLA), pelargonidin (PEL) and Epigallocatechin gallate (EGCG) | Chemoprevention of genotoxic damage in HL-60 cells. | The single compounds showed protection (at low concentrations) against genotoxic activity, which was neither additive nor synergistic. 5–50 µM CLA, 0.05–0.5 µM PEL, 0.5–5 µM EGCG and 0.01–0.1 µM Resv | [12] |
| Quercetin and Caffeic acid (CAF) | Various antioxidant/scavenging assays. | No clear additive nor synergistic effects were observed. | [13] |
| Quercetin (QUE) | Cell proliferation and cell viability in colon HT-29 cells | Dose-dependent effect of the mixture of Resv and QUE (0 to 60 µg/mL totally). The results were not compared with the single compounds. | [14] |
| Quercetin (QUE) and Caffeic acid (CAF) | Anti-amyloid activities in hen egg white lysozymes. | Both synergetic or antagonistic effects were observed by these combinations while testing 10 pM to 1 mM equimolar combinations of Resv, QUE and CAF. | [15] |
| Gallic acid, caffeic acid, catechin, quercetin | Different antioxidant assays. | A synergistic effect was observed for catechin and Resv.  Other combinations gave diverse responses dependent on the antioxidant test applied. | [16] |
| Equol | Mitochondrial function in HUVEC cells. | Combination of equol (10 µM) and Resv (10 µM) was more effective in increasing the mitochondrial labelling than Resv alone | [17] |
| Grape Seed Extract (GSE) | Cell proliferation assay of HCT-116 cells, apoptosis. | Resv (25 µM) potentiates GSE (35 µg/mL) induced apoptosis and suppressed colon cancer cell prolferation. | [18] |
| Capsaicin | Nitric oxide (NO•) induction in A375 cells. | Capsaicin (100 µM) and Resv (50 µM), alone or in combination, inhibited cell growth, promoted apoptosis and the level of NO•. Additive effect. | [19] |
| Conjugated linoleic acid (CLA) | Modulation of triglyceride (TG) accumulation in mature adipocytes (3T3-L1 cells) | Separately, Resv (10 or 100 µM) and CLA (10 or 100 µM) significantly reduced the cellular TG level, but their combination did not add further effect. | [20] |
| Docosahexaenoic acid (DHA) or eicosapen-taenoic acid (EPA) | Anti-inflammatory effects in murine macrophages *in vitro* (Raw 264.7 cells) | Both Resv (2.5 µg/mL) + EPA (30 µM) and Resv (2.5 µg/mL) + DHA (30 µM) had an enhanced anti-inflammatory effect, in comparison with EPA, DHA and Resv alone. | [21] |
| Rapamycin (Rapa) | Cell viability of breast cancer cell lines (MDA-MB-231 and MCF-7). | A stronger effect on cell number was observed by the combinatory treatment by Resv (10–50 µM) and Rapa (1–10,000 nM), compared with the single compounds | [22] |
| β-hydroxy-β-methyl butyrate (HMB) or leucine | Fat metabolism, AMPK and Sirt1 activity in muscle (C2C12) or adipocyte (3T3-L1) cells. | Fatty acid oxidation, AMPK and Sirt1 activities were significantly increased by the combinations of Resv (200 nM) with HMB (5 µM) or leucine (0.5 mM). | [23,24] |
| Crocin (CRN) | Light-induced oxidative damage in human RPE cells | The combination of CRN (100 µM) with RSV (1 µM) showed synergistic cytoprotective effect in relation to cell viability. | [25] |
| Sulfated (1→3)-α-L-fucan (ScF2) | Cell viability and apoptosis in the HCT 116 human colon cancer cell | ScF2 (100–300 µg/mL) potentiateed the Resv-induced (40 µM) reduction in cell viability and Resv-induced apoptosis. | [26] |
| 1α,25-dihydroxyvitamin D_3_ (1α,25(OH)_2_D_3_) | Induction of the cathelicidin antimicrobial peptide (CAMP) gene. | CAMP gene expression is synergistically activated by combination of Resv (10 µM) and 1α, 25(OH)_2_D_3_ (10 nM). | [27] |
| Dimethylsphingosine (DMS) | Viability of human gastric cancer cells (HT-29, SNU-1 and SNU-668). | Combination of Resv (50 and 100 µM) and DMS (5 and 10 µM) increased cytotoxicity. | [28] |

**Table S4.** Effects of various naturally occurring compounds *in vivo* in combination with resveratrol (Resv).

| **Compounds** | **Activity** | **Effect** | **Reference** |
| --- | --- | --- | --- |
| γ-tocotrienol | Ischemia/reperfusion in rats | Greater degree of cardioprotection their combination than the single compound (2.5 mg/kg Resv for 15 days and/or 0.3 mg/kg γ-tocotrienol for 30 days).  No test for synergism is shown even that synergy is stated. | [29] |
| Vitamin E, vitamin C and α-lipoic acid | Lindane-induced toxicity in mice olfactory lobe and cerebrum | The combination counteracted the lindande-induced toxicity. No test of the combinatory effect in contrast to the effects of the single compounds. The mix contains 50 mg vitamin E/kg, 50 mg vitamin C/kg, 20 mg α-lipoic acid /kg and 5 mg Resv/kg b.w. | [30] |
| Mix of Resv, Vitamin C, α-lipoic acid and vitamin E | Lindane-induced toxicity in the cerebellum and pons-medulla oblongata of Swiss mice | The combination counteracted the lindane-induced toxicity. No test of the combinatory effect in contrast to the effects of the single compounds. The mix contains 50 mg vitamin E/kg, 50 mg vitamin C/kg, 20 mg α-lipoic acid /kg and 5 mg Resv/kg b.w. | [31] |
| Lipoic Acid (LA) | Modulation of infarct volume after middle cerebral artery and reperfusion in rats | Pretreatment with non-neuroprotective doses of Resv (2 × 10^−6^ mg/kg i.v.) and lipoic acid (LA; 0.005 mg/kg) in combination produced significant neuroprotection as found by Resv (2 × 10^−3^ mg/kg i.v.) alone. | [32] |
| Conjugated linoleic acid (CLA) | Modulation of body fat in rats (male Wistar) fed an obesogenic diet | Separately, Resv (30 mg/kg b.w./day in the diet) and CLA (0.5% in the diet) significantly reduced body fat but not when combined. Resv, as well as the combination Resv + CLA, improved glycaemic control. | [33] |
| Ellagic acid (EA), calcium d-glucarate (CG) and grape extract (GE) | Chemically (DMBA)-induced skin tumorigenesis in SENCAR mice | Stronger effect of the combinations of Resv (topical, 2.5 or 5 μmol/dose) and either EA (1%–4% in the diet) or CG (1%–4% in the diet) or GE (topical, 1 or 2.5 μmol/dose) than the single compounds. These were found synergistically active in several cases. | [34] |
| Calcium d-glucarate (CG) and ursolic acid (UA) | 7,12-dimethylbenz[a]anthra-cene (DMBA)-initiated, 12-*O*-tetradecanoylphorbol-13-acetate (TPA)-promoted multi-stage skin carcinogenesis model in SENCAR mice. | Epidermal cell proliferation was only reduced when UA (topical application of 1 µmol) was applied alone or in combination with CG (2% in diet, given 2 weeks before initiation). No effect of Resv (topical application of 2.5 µmol). | [35] |
| Ursolic acid (UA) | Epidermal hyperproliferation, skin inflammation, inflamma-tory gene expression and skin tumor promotion in TPA-exposed mouse skin | Epidermal hyperproliferation and pro-inflammatory cytokines were reduced more with UA + Resv compared with the single compounds.  Greater inhibition of tumor multiplicity and tumor size with the combination than either agent alone. | [36] |
| Curcumin (CUR) | Benzo[a]pyrene-induced micronuclei formation and apoptosis | CUR (by gavage, 60 mg/kg b.w., three times/week) and Resv (5.7 µg/mL in drinking water, three times/week) alone did reduced micronuclei formation, but not significantly. A significant reduction was observed by the combination of CUR and Resv. The combination did also improve the number of apoptotic cells. | [37] |
| Curcumin (CUR) | Tumor burden in mice of exposure to benzo[a]pyrene | CUR (by gavage, 60 mg/kg b.w., three times/week) reduced the tumor burden and combined with Resv (5.7 µg/mL in drinking water, three times /week), a further reduction was observed. Various biomarkers were found to be modulated significantly by the Cur and Resv combination | [38] |
| Vitamin D, genistein (GEN), and quercetin (QUE) | Inhibition of bone loss and decreased adiposity. | Aged, ovariectomized female rats supplemented with vitamin D (2400 IU/kg b.w.) combined with QUE (80, 400, or 2000 mg/kg of diet), GEN (64, 256, or 1040 mg/kg of diet), and Resv (16, 80, or 400 mg/kg of diet) for eight weeks improved bone mineral density and reduced body weight gain, and a significantly decreased bone marrow adipocytes. | [39] |
| Quercetin (QUE) | Reduction of restenosis in female B6.129 mice. | Resv (250 mg/kg diet, for two weeks) decreased stenosis (intima:media ratio), but QUE (60 mg/kg diet, for two weeks) alone had no effect. It potentiated the effect of Resv treatment stronger than Resv alone. | [40] |
| Silymarin (SIL)-containing extract | Tumor frequency in livers of HBx transgenic male mice (C57BL/6 background). | Combination of SIL-containing extract (35% SIL) and Resv-containing extract (20% Resv) caused a much stronger tumor reduction compared with Resv or SIL alone. | [41] |
| Β-glucan and vitamin C | Reduction of tumor weight after injections of Ptas64 mammary carcinoma cells or Lewis lung carcinoma cells promoted by cyclophospha-mide. | In both models, statistically significant reduced tumor weight was observed by the combinations of two or three compounds and by β-glucan alone. Each compound (100 μg) was applied i.p. once daily for 14 days. | [42] |
| Zn ions | Chemoprevention effects on DMBA-induced mammary cancer in rats | The DMBA-induced mammary cancer response was not inhibited and the combination of Zn (231 mg Zn/kg diet) + Resv (0.2 mg/kg b.w.) did have a faster onset of the DMBA-induced carcinogenesis. | [43] |
| Hydroxymethyl- butyrate (HMB) or leucine | Insulin sensitivity and inflammatory biomarker responses in diet-induced obese (DIO) mice | Even low Resv (12.5 mg/kg diet) in combination with HMB (2 g/kg diet) and leucine (24 g/kg diet) caused an increased insulin sensitivity and improved inflammatory stress biomarkers. Such response was not observed with high Resv alone (225 mg Resv/kg diet) | [23] |
| Melatonin | *N*-methyl-*N*-nitrosourea  (NMU)-induced mammary carcinogenesis in female Sprague-Dawley rats. | The combination of Resv (100 mg/kg diet) and melatonin (20 mg /L in drinking water) significantly reduced the tumor incidence, with none or minor effects of the two compounds given alone. | [44] |
| Melatonin | Obesity and precipitated metabolic disturbances following ovariectomy and fructose diet in female Sprague-Dawley rats. | Ovariectomy caused an increase in body weight, body mass index, feed efficiency, serum glucose, levels of cholesterol, triglycerides, and free fatty acids, which was further exacerbated by fructose diet. These parameters were significantly decreased by Resv (50 mg/kg/day p.o.), alone and in combination with melatonin (3 mg/kg/day p.o.). | [45] |
| Rapamycin | Insulin sensitivity in obese male mice (C57BL/6NCr strain). | High-fat diet induced insulin level was only significantly decreased by the combination of Resv (200 mg/kg b.w. by gavage) and rapamycin (1.5 mg/kg b.w. by gavage). | [46] |
| Human Studies | | | |
| Calcium fructoborate | Angina episodes and inflammatory responses in 87 subjects, 60-days treatment. | hsCRP reduction was strongest by calcium fructoborate alone (112 mg/day). The decrease in the number of angina episodes was the strongest by the combination of Resv (20 mg/day) and calcium fructoborate. | [47] |
| Quercetin (QUE) | Exercise-induced lipid peroxidation in 14 athletes. Treatment for 6 days + 1 day before exercise. | Combination of Resv and QUE significantly reduced exercise-induced lipid peroxidation without associated changes in inflammation or plasma anti-oxidant status. Resv and QUE combination (120 mg Rsv and 225 mg QUE) for 6 days and 240 mg Resv + 450 mg QUE on day 7, just prior to exercise compared with placebo. | [48] |

References

1. Mikstacka, R.; Rimando, A.M.; Ignatowicz, E. Antioxidant effect of *trans*-resveratrol, pterostilbene, quercetin and their combinations in human erythrocytes *in vitro*. *Plant Foods Hum. Nutr.* **2010**, *65*, 57–63.
2. Ravagnan, G.; de Filippis, A.; Carteni, M.; de Maria, S.; Cozza, V.; Petrazzuolo, M.; Tufano, M.A.; Donnarumma, G. Polydatin, a natural precursor of resveratrol, induces beta-defensin production and reduces inflammatory response. *Inflammation* **2013**, *36*, 26–34.
3. Aftab, N.; Likhitwitayawuid, K.; Vieira, A. Comparative antioxidant activities and synergism of resveratrol and oxyresveratrol. *Nat. Prod. Res.* **2010**, *24*, 1726–1733.
4. Aftab, N.; Vieira, A. Antioxidant activities of curcumin and combinations of this curcuminoid with other phytochemicals. *Phytother. Res.* **2010**, *24*, 500–502.
5. Iwuchukwu, O.F.; Tallarida, R.J.; Nagar, S. Resveratrol in combination with other dietary polyphenols concomitantly enhances antiproliferation and UGT1A1 induction in Caco-2 cells. *Life Sci.* **2011**, *88*, 1047–1054.
6. Du, Q.; Hu, B.; An, H.M.; Shen, K.P.; Xu, L.; Deng, S.; Wei, M.M. Synergistic anticancer effects of curcumin and resveratrol in Hepa1–6 hepatocellular carcinoma cells. *Oncol. Rep.* **2013**, *29*, 1851–1858.
7. Mohapatra, P.; Satapathy, S.R.; Siddharth, S.; Das, D.; Nayak, A.; Kundu, C.N. Resveratrol and curcumin synergistically induces apoptosis in cigarette smoke condensate transformed breast epithelial cells through a p21(Waf1/Cip1) mediated inhibition of Hh-Gli signaling. *Int. J. Biochem. Cell Biol.* **2015**, *66*, 75–84.
8. Jiang, H.; Shang, X.; Wu, H.; Huang, G.; Wang, Y.; Al-Holou, S.; Gautam, S.C.; Chopp, M. Combination treatment with resveratrol and sulforaphane induces apoptosis in human U251 glioma cells. *Neurochem. Res.* **2010**, *35*, 152–161.
9. Masuelli, L.; Marzocchella, L.; Focaccetti, C.; Tresoldi, I.; Palumbo, C.; Izzi, V.; Benvenuto, M.; Fantini, M.; Lista, F.; Tarantino, U.; *et al.* Resveratrol and diallyl disulfide enhance curcumin-induced sarcoma cell apoptosis. *Front. Biosci.* **2012**, *17*, 498–508.
10. Kurin, E.; Atanasov, A.G.; Donath, O.; Heiss, E.H.; Dirsch, V.M.; Nagy, M. Synergy study of the inhibitory potential of red wine polyphenols on vascular smooth muscle cell proliferation. *Planta Med.* **2012**, *78*, 772–778.
11. Dhandayuthapani, S.; Marimuthu, P.; Hormann, V.; Kumi-Diaka, J.; Rathinavelu, A. Induction of apoptosis in HeLa cells via caspase activation by resveratrol and genistein. *J. Med. Food* **2013**, *16*, 139–146.
12. Abraham, S.K.; Eckhardt, A.; Oli, R.G.; Stopper, H. Analysis of *in vitro* chemoprevention of genotoxic damage by phytochemicals, as single agents or as combinations. *Mutat. Res.* **2012**, *744*, 117–124.
13. Kurin, E.; Mucaji, P.; Nagy, M. *In vitro* antioxidant activities of three red wine polyphenols and their mixtures: An interaction study. *Molecules* **2012**, *17*, 14336–14348.
14. Del Follo-Martinez, A.; Banerjee, N.; Li, X.; Safe, S.; Mertens-Talcott, S. Resveratrol and quercetin in combination have anticancer activity in colon cancer cells and repress oncogenic microRNA-27a. *Nutr. Cancer* **2013**, *65*, 494–504.
15. Gazova, Z.; Siposova, K.; Kurin, E.; Mucaji, P.; Nagy, M. Amyloid aggregation of lysozyme: The synergy study of red wine polyphenols. *Proteins* **2013**, *81*, 994–1004.
16. Skroza, D.; Mekinic, I.G.; Svilovic, S.; Simar, V.; Katalinic, V. Investigation of the potential synergistic effect of resveratrol with other phenolic compounds: A case of binary phenolic mixtures. *J. Food Compost. Anal.* **2015**, *38*, 13–18.
17. Davinelli, S.; Sapere, N.; Visentin, M.; Zella, D.; Scapagnini, G. Enhancement of mitochondrial biogenesis with polyphenols: Combined effects of resveratrol and equol in human endothelial cells. *Immun. Ageing* **2013**, *10*, 28.
18. Radhakrishnan, S.; Reddivari, L.; Sclafani, R.; Das, U.N.; Vanamala, J. Resveratrol potentiates grape seed extract induced human colon cancer cell apoptosis. *Front. Biosci.* **2011**, *3*, 1509–1523.
19. Kim, M.Y. Nitric oxide triggers apoptosis in A375 human melanoma cells treated with capsaicin and resveratrol. *Mol. Med. Rep.* **2012**, *5*, 585–591.
20. Lasa, A.; Miranda, J.; Churruca, I.; Simon, E.; Arias, N.; Milagro, F.; Martinez, J.A.; Portillo, M.D. The combination of resveratrol and CLA does not increase the delipidating effect of each molecule in 3T3-L1 adipocytes. *Nutr. Hosp.* **2011**, *26*, 997–1003.
21. Pallares, V.; Calay, D.; Cedo, L.; Castell-Auvi, A.; Raes, M.; Pinent, M.; Ardevol, A.; Arola, L.; Blay, M. Enhanced anti-inflammatory effect of resveratrol and EPA in treated endotoxin-activated RAW 264.7 macrophages. *Br. J. Nutr.* **2012**, *108*, 1562–1573.
22. He, X.; Wang, Y.; Zhu, J.H.; Orloff, M.; Eng, C. Resveratrol enhances the anti-tumor activity of the mTOR inhibitor rapamycin in multiple breast cancer cell lines mainly by suppressing rapamycin-induced AKT signaling. *Cancer Lett.* **2011**, *301*, 168–176.
23. Bruckbauer, A.; Zemel, M.B.; Thorpe, T.; Akula, M.R.; Stuckey, A.C.; Osborne, D.; Martin, E.B.; Kennel, S.; Wall, J.S. Synergistic effects of leucine and resveratrol on insulin sensitivity and fat metabolism in adipocytes and mice. *Nutr. Metab.* **2012**, *9*, 77.
24. Bruckbauer, A.; Zemel, M.B. Synergistic Effects of Polyphenols and Methylxanthines with leucine on AMPK/Sirtuin-mediated metabolism in muscle cells and adipocytes. *PLoS ONE* **2014**, *9*, e89166.
25. Kernt, M.; Piraee, M.; Wertheimer, C.; Haritoglou, C.; Ulbig, M.W.; Kampik, A. Synergistic cytoprotective effect of crocin (CRN) and resveratrol (RSV) on primary human retinal pigmentepithelium (RPE) cells. *Planta Med.* **2012**, *78*, 1115.
26. Vishchuk, O.S.; Ermakova, S.P.; Zvyagintseva, T.N. The effect of sulfated (1→3)-alpha-l-fucan from the brown alga Saccharina cichorioides Miyabe on resveratrol-induced apoptosis in colon carcinoma Cells. *Mar. Drugs* **2013**, *11*, 194–212.
27. Guo, C.; Sinnott, B.; Niu, B.; Lowry, M.B.; Fantacone, M.L.; Gombart, A.F. Synergistic induction of human cathelicidin antimicrobial peptide gene expression by vitamin D and stilbenoids. *Mol. Nutr. Food Res.* **2014**, *58*, 528–536.
28. Shin, K.O.; Park, N.Y.; Seo, C.H.; Hong, S.P.; Oh, K.W.; Hong, J.T.; Han, S.K.; Lee, Y.M. Inhibition of sphingolipid metabolism enhances resveratrol chemotherapy in human gastric cancer cells. *Biomol. Ther.* **2012**, *20*, 470–476.
29. Lekli, I.; Ray, D.; Mukherjee, S.; Gurusamy, N.; Ahsan, M.K.; Juhasz, B.; Bak, I.; Tosaki, A.; Gherghiceanu, M.; Popescu, L.M.; *et al.* Co-ordinated autophagy with resveratrol and gamma-tocotrienol confers synergetic cardioprotection. *J. Cell. Mol. Med.* **2010**, *14*, 2506–2518.
30. Bano, M.; Bhatt, D.K. Ameliorative effect of a combination of vitamin E, vitamin C, alpha-lipoic acid and stilbene resveratrol on lindane induced toxicity in mice olfactory lobe and cerebrum. *Indian J. Exp. Biol.* **2010**, *48*, 150–158.
31. Bist, R.; Bhatt, D.K. Augmentation of cholinesterases and ATPase activities in the cerebellum and pons-medulla oblongata, by a combination of antioxidants (resveratrol, ascorbic acid, alpha-lipoic acid and vitamin E), in acutely lindane intoxicated mice. *J. Neurol. Sci.* **2010**, *296*, 83–87.
32. Saleh, M.C.; Connell, B.J.; Rajagopal, D.; Khan, B.V.; Abd-El-Aziz, A.S.; Kucukkaya, I.; Saleh, T.M. Co-administration of resveratrol and lipoic acid, or their synthetic combination, enhances neuroprotection in a rat model of ischemia/reperfusion. *PLoS ONE* **2014**, *9*, e87865.
33. Arias, N.; Macarulla, M.T.; Aguirre, L.; Martinez-Castano, M.G.; Gomez-Zorita, S.; Miranda, J.; Martinez, J.A.; Portillo, M.P. The combination of resveratrol and conjugated linoleic acid is not useful in preventing obesity. *J. Physiol. Biochem.* **2011**, *67*, 471–477.
34. Kowalczyk, M.C.; Kowalczyk, P.; Tolstykh, O.; Hanausek, M.; Walaszek, Z.; Slaga, T.J. Synergistic effects of combined phytochemicals and skin cancer prevention in SENCAR mice. *Cancer Prev. Res.* **2010**, *3*, 170–178.
35. Kowalczyk, M.C.; Junco, J.J.; Kowalczyk, P.; Tolstykh, O.; Hanausek, M.; Slaga, T.J.; Walaszek, Z. Effects of combined phytochemicals on skin tumorigenesis in SENCAR mice. *Int. J. Oncol.* **2013**, *43*, 911–918.
36. Cho, J.; Rho, O.; Junco, J.; Carbajal, S.; Siegel, D.; Slaga, T.J.; DiGiovanni, J. Effect of combined treatment with Ursolic acid and resveratrol on skin tumor promotion by 12-*O*-tetradecanoylphorbol-13-acetate. *Cancer Prev. Res.* **2015**, *8*, 817–825.
37. Malhotra, A.; Nair, P.; Dhawan, D.K. Curcumin and resveratrol in combination modulates benzo(a)pyrene-induced genotoxicity during lung carcinogenesis. *Hum. Exp. Toxicol.* **2012**, *31*, 1199–1206.
38. Malhotra, A.; Nair, P.; Dhawan, D.K. Study to evaluate molecular mechanics behind synergistic chemo-preventive effects of curcumin and resveratrol during lung carcinogenesis. *PLoS ONE* **2014**, *9*, e93820.
39. Lai, C.Y.; Yang, J.Y.; Rayalam, S.; Della-Fera, M.A.; Ambati, S.; Lewis, R.D.; Hamrick, M.W.; Hartzell, D.L.; Baile, C.A. Preventing bone loss and weight gain with combinations of vitamin D and phytochemicals. *J. Med. Food* **2011**, *14*, 1352–1362.
40. Khandelwal, A.R.; Hebert, V.Y.; Kleinedler, J.J.; Rogers, L.K.; Ullevig, S.L.; Asmis, R.; Shi, R.; Dugas, T.R. Resveratrol and quercetin interact to inhibit neointimal hyperplasia in mice with a carotid injury. *J. Nutr.* **2012**, *142*, 1487–1494.
41. Hsieh, W.C.; Yang, C.W.; Haung, Y.S.; Chao, T.W.; Tsai, T.F.; Su, I.J. Chemoprevention of HBV-related hepatocellular carcinoma by the combined product of resveratrol and silymarin in transgenic mice. *Funct. Foods Health Dis.* **2013**, *3*, 341–352.
42. Vetvicka, V.; Vetvickova, J. Combination of glucan, resveratrol and vitamin C demonstrates strong anti-tumor potential. *Anticancer Res.* **2012**, *32*, 81–87.
43. Bobrowska-Korczak, B.; Skrajnowska, D.; Tokarz, A. The effect of dietary zinc- and polyphenols intake on DMBA-induced mammary tumorigenesis in rats. *J. Biomed. Sci.* **2012**, *19*, 43.
44. Kiskova, T.; Ekmekcioglu, C.; Garajova, M.; Orendas, P.; Bojkova, B.; Bobrov, N.; Jager, W.; Kassayova, M.; Thalhammer, T. A combination of resveratrol and melatonin exerts chemopreventive effects in *N*-methyl-*N*-nitrosourea-induced rat mammary carcinogenesis. *Eur. J. Cancer Prev.* **2012**, *21*, 163–170.
45. Majumdar, A.S.; Giri, P.R.; Pai, S.A. Resveratrol- and melatonin-abated ovariectomy and fructose diet-induced obesity and metabolic alterations in female rats. *Menopause* **2014**, *21*, 876–885.
46. Leontieva, O.V.; Paszkiewicz, G.; Demidenko, Z.N.; Blagosklonny, M.V. Resveratrol potentiates rapamycin to prevent hyperinsulinemia and obesity in male mice on high fat diet. *Cell Death Dis.* **2013**, *4*, e472.
47. Militaru, C.; Donoiu, I.; Craciun, A.; Scorei, I.D.; Bulearca, A.M.; Scorei, R.I. Oral resveratrol and calcium fructoborate supplementation in subjects with stable angina pectoris: Effects on lipid profiles, inflammation markers, and quality of life. *Nutrition* **2013**, *29*, 178–183.
48. McAnulty, L.S.; Miller, L.E.; Hosick, P.A.; Utter, A.C.; Quindry, J.C.; McAnulty, S.R. Effect of resveratrol and quercetin supplementation on redox status and inflammation after exercise. *Appl. Physiol. Nutr. Metab.* **2013**, *38*, 760–765.
